# Design Challenges in Polymeric Scaffolds for Tissue Engineering

**DOI:** 10.3389/fbioe.2021.617141

**Published:** 2021-06-14

**Authors:** Maria I. Echeverria Molina, Katerina G. Malollari, Kyriakos Komvopoulos

**Affiliations:** Department of Mechanical Engineering, University of California, Berkeley, Berkeley, CA, United States

**Keywords:** scaffolds, tissue engineering, cells, biopolymers, structure, biochemistry, biodegradability, mechanical behavior

## Abstract

Numerous surgical procedures are daily performed worldwide to replace and repair damaged tissue. Tissue engineering is the field devoted to the regeneration of damaged tissue through the incorporation of cells in biocompatible and biodegradable porous constructs, known as scaffolds. The scaffolds act as host biomaterials of the incubating cells, guiding their attachment, growth, differentiation, proliferation, phenotype, and migration for the development of new tissue. Furthermore, cellular behavior and fate are bound to the biodegradation of the scaffold during tissue generation. This article provides a critical appraisal of how key biomaterial scaffold parameters, such as structure architecture, biochemistry, mechanical behavior, and biodegradability, impart the needed morphological, structural, and biochemical cues for eliciting cell behavior in various tissue engineering applications. Particular emphasis is given on specific scaffold attributes pertaining to skin and brain tissue generation, where further progress is needed (skin) or the research is at a relatively primitive stage (brain), and the enumeration of some of the most important challenges regarding scaffold constructs for tissue engineering.

## Introduction

The human body is by far the most sophisticated autonomous system consisting of billions of molecular nanomachines built from the DNA code of a person's zygote, which not only can renew certain type of cells in a complex programmable manner but also repair damaged tissue. However, this self-healing capacity is limited to the type of cells comprising the tissue and the multiple factors controlling tissue self-repair, including degenerative diseases, traumatic events, and age. In fact, some tissues do not exhibit a self-healing capability. To this end, tissue engineering (TE) plays a vital role in the development of functional constructs that can promote the generation of tissue and organ equivalents, or enhance tissue regeneration to restore and/or improve the functionality of damaged tissues and organs, ultimately replacing autografts, allografts, or even whole organs. Examples of engineered tissue applications include skin, cartilage, veins, arteries, brain, gastrointestinal tissues, and cornea. Although some of the former engineered tissues have been approved for human use, successful transplantation and, especially, implant longevity are limited due to complications leading to biomaterial rejection by various complex mechanisms (Sheikh et al., 2015; Anderson and Jiang, 2017).

The increasing demand for human tissues and organs highlighted in a recent report (Israni et al., 2020) and the acceleration of deaths due to acute illnesses, such as ischemic coronary diseases (World Health Organization, 2018), have increased the market demand for TE (Grand View Research, 2020), highlighting the importance of tissue regeneration and repair. TE is a multidisciplinary field concerned with the creation of tissue by different techniques that depend on cell seeding of non-woven, porous scaffold biomaterials and the incorporation of various growth factors that promote tissue growth. Scaffolds are 3D constructs that mimic the extracellular matrix (ECM) of native tissue, typically made of biodegradable and biocompatible polymers. A polymeric scaffold material must be characterized by multiple attributes, such as no chronic inflammatory response and/or rejection by the surrounding tissue, degradation rate that matches the healing or growth rate of the tissue, non-toxic byproducts that can be expelled by the body in a timely fashion, acceptable shelf life, good mechanical properties compatible with those of the native ECM, which do not impede cell interaction, and no changes in architecture and properties due to sterilization (Pruitt and Chakravartula, 2011). However, cell culture, tissue growth, and implantation may affect the scaffold properties and performance due to material degradation and ever-changing interfaces. The scaffold performance is quantified in multiple ways, especially by the cell behavior that it can stimulate and how closely it mimics the native ECM of the generated tissue.

Important developments in TE have been encountered in the past two decades, largely due to significant advances in biomaterials, fabrication, and techniques designed to instigate specific cellular behaviors that stimulate the formation of tissue analogs. Biomaterials showing a versatile performance depending on the biological environment have been developed by modifying their chemistry. Examples include the assimilation of specific surface cues by plasma treatment, layer-by-layer deposition or surface functionalization with polydopamine (PDA), and the so-called “click” chemistry, which can impart specificity for multiple cues (e.g., drug delivery, crosslinking, and cell attachment) to the bulk and the surface of polymeric scaffolds. Hydrogels are a special class of materials that have attracted considerable attention as scaffolds because they can be delivered to the desired site by minimally invasive methods (e.g., by injection) and doped with drugs, cells, and growth factors, therefore showing high potential for many TE applications, such as brain and skin tissues. Notable breakthroughs in TE have also been accounted as a result of significant progress in the development of versatile fabrication methods for scaffold engineering (Dutta et al., 2017; Walker and Santoro, 2017; Zhao et al., 2018). For example, 3D printing techniques have provided capabilities to create specific and controlled geometries and shapes of constructs and to simultaneously use biomaterials, growth factors, and cells to create composites that exhibit unique degradation rates, enhanced cell differentiation, specific biological cues, and excellent biomechanical properties throughout the scaffold's lifetime (Do et al., 2015; Di Luca et al., 2016c; Bittner et al., 2018; Ng et al., 2018; Han et al., 2021). Other less common and/or new fabrication techniques, such as multiphoton, laser-assisted, and computed axial lithography, have also been used to create scaffolds for TE (Liang et al., 2021). In addition, conventional methods modified to incorporate cells and growth factors in polymeric constructs, such as electrospinning and gas foaming, have gained attention recently (Costantini and Barbetta, 2018; Hong et al., 2019; Karpov et al., 2020).

The understanding of the function of different cells that form a tissue or an organ, the improved techniques for harvesting cells, and the insight into how the healing multifactorial process works as well as the interplay of the physicochemical properties and morphology of biomaterials have led to the development of scaffolds that can stimulate the desired cellular behavior, while providing the chemical and mechanical cues for achieving tissue growth and regeneration. However, the difficulty to reproduce the complex biological environment during *in vitro* testing inhibits direct comparisons with the material's behavior *in vivo*. Developments in bioreactors have enabled the incorporation of *in vivo*-like signals, such as static and dynamic electromechanical cues and control of the pH, temperature, gene expression, and growth factors, which are closely associated with the initial stages of cell differentiation. More importantly, because most of the engineered tissues are small versions of human counterparts, they can only be tested in animal models, with most of the attempts to create full size organs being largely ineffective with only a few exceptions, such as 3D printed human ears (Zhou et al., 2018). Nevertheless, some of the engineered tissues (e.g., skin, cornea, and vascular grafts) have reached pre-clinical trials or commercial application, despite the fact that they cannot be implanted to all patients and/or fully restore normal tissue function, thus illuminating the complexity to recreate totally functional tissues. However, despite significant progress in the development of organ-on-chip platforms that can more closely reproduce physiological settings *in vitro* (Zhang et al., 2018; Wu et al., 2020), animal models are still a fundamental part of the research needed to better understand diseases and to develop more effective drug delivery methods.

Because scaffold design can greatly affect cellular behavior from its inception, there are many critical factors to consider in TE. The objective of this article is to provide an appraisal of the current state-of-the-art in TE, specifically focusing on the correlation of scaffold parameters and cell fate, the types of polymeric materials currently used in scaffold engineering, and the effects of biochemical characteristics, structure architecture, biodegradability, and mechanical behavior of scaffold materials on the resulting biomimicry cues and their ability to stimulate specific cell behaviors. In addition, two important soft tissue application areas, namely skin and brain scaffold engineering, are discussed in the context of associated anatomy and biological functions, and the design challenges of scaffolds intended for these TE applications are interpreted in the light of recent advances in scaffold engineering.

## Scaffold Main Characteristics

Scaffolds play a fundamental role in TE because they provide mechanical support, allow perfusion of nutrients and oxygen, transfer biochemical signals that modulate cell behavior (e.g., attachment, motility, proliferation, and differentiation), and can be used to release drugs and growth factors. A scaffold must mimic the ECM by exhibiting the biological, chemical, and mechanical cues that influence cell phenotype and tissue formation. Biomaterials for tissue generation must exhibit tailorable properties to enhance cell attachment, migration, growth, and differentiation, prevent undesirable host responses that lead to chronic inflammation at the biomaterial interface, display chemical and mechanical stability for providing structural support while demonstrating controllable microstructure and adequate porosity, and show good biodegradability without producing toxic residues and byproducts. Biomaterial scaffolds for TE must be developed according to the aforementioned design criteria by integrating engineering and biological principles and molecular cues that imitate critical structural aspects of the native tissue and can effectively direct cellular behavior and functionality. Based on the preceding requirements, biomaterial scaffolds can be classified into three main material categories, namely architectured materials with fibrous and porous structures, hybrid or composite materials, and hydrogels.

Architectured materials are mainly synthetic biodegradable polymers characterized by 3D fibrous networks. Natural ECM decellularized tissue materials (typically powdered) also belong in this category. These materials can be formed by various fabrication techniques, such as electrospinning, freeze-drying, gas foaming, solvent casting, particulate or porogen leaching, phase separation, self-assembly, and 3D printing. Polymers that belong in this category are synthetic biodegradable polymers, such as poly(lactic acid) (PLA), poly(glycolic acid) (PGA), poly(ε-caprolactone) (PCL), polyvinyl alcohol (PVA), polyethylene glycol (PEG), polyethylene oxide (PEO), polyurethane (PU), and polyvinylpyrrolidone.

Hybrid materials encompass blends of synthetic polymers, natural polymers, and decellularized tissue with one or few solvents. Alternatively, composite materials include the same polymer-based constituents as hybrid materials, but possess either layered or multi-material structures made by various processes. Hybrid and composite materials are typically produced by fabrication methods designed to synthesize architectured biomaterials, with a few exceptions, such as hydrogel-based hybrid materials.

Hydrogels are swollen natural or synthetic polymer networks crosslinked by physical and/or covalent bonds, which show a high potential for tissue repair because they form ECM-like architectures and can serve as platforms for minimally invasive delivery of macromolecules to the injury site (Hsieh et al., 2017; Chen et al., 2019; Peppas and Hoffman, 2020). Polymer-based hydrogels show numerous advantages over architectured and hybrid/composite materials, such as different chemical, mechanical, and spatial cues for encapsulating cells, and can provide bioactive signals to the host tissue. The capability of hydrogels to modulate nutrient diffusion and cell motility and their mechanical stability depend on the crosslink density and mesh size, which can be tuned during the fabrication.

**Figure 1:**
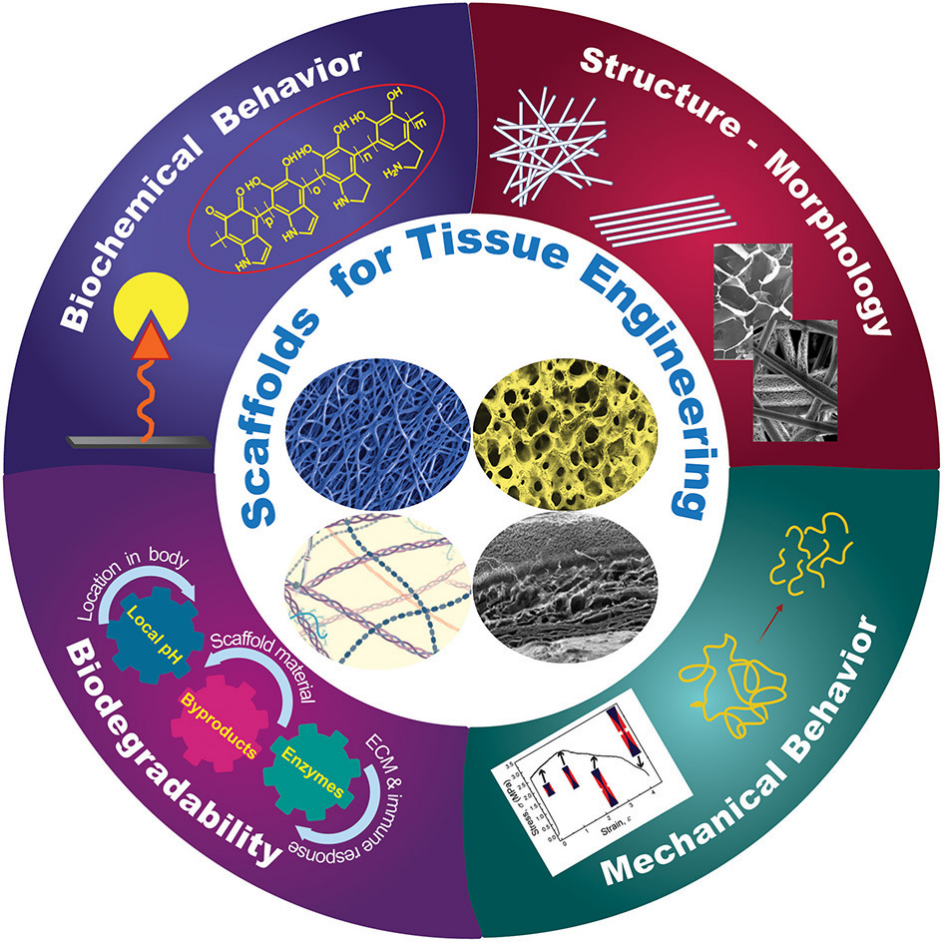
Schematic representation of different design characteristics of biomaterial scaffolds that are necessary for eliciting the cellular behavior required for a specific tissue application. Effective tuning of the parameters described in each category plays a critical role in the performance of architectured (fibrous and porous), hybrid, and hydrogel scaffolds for tissue generation and repair.

Basic understanding of how a scaffold can be modified to elicit the cellular response needed for a specific TE application is of paramount importance in scaffold engineering and requires insight into the effects of the biochemical characteristics, structure and morphology, biodegradability, and mechanical behavior on the scaffold performance. The main design characteristics of scaffolds intended for the TE applications discussed in this section are depicted in Figure 1.

### Biochemical Behavior

The affinity of the cells for a scaffold greatly depends on the chemical and topological surface and bulk cues of the scaffold material. Specifically, the hydrophilicity, charge density, and chemical specificity of the scaffold surface affect cell attachment, whereas the bulk chemical characteristics influence cell signaling and infiltration. Both surface and bulk chemistry synergistically regulate cell growth, migration, differentiation, ECM synthesis, and tissue morphogenesis and are critical to achieving scaffold biocompatibility. Scaffold chemical modification has emerged as an effective means of producing biochemical specificity and recognition, with chemical moieties incorporated in the bulk and/or surface of the scaffolds to mediate cell behavior and functionality, direct inflammatory and immunological response, and ameliorate foreign body reaction at the scaffold-tissue interface (Allen et al., 2006; Anderson et al., 2008; Owen and Shoichet, 2010; Nimmo et al., 2011; Lanza et al., 2020).

**Figure 2:**
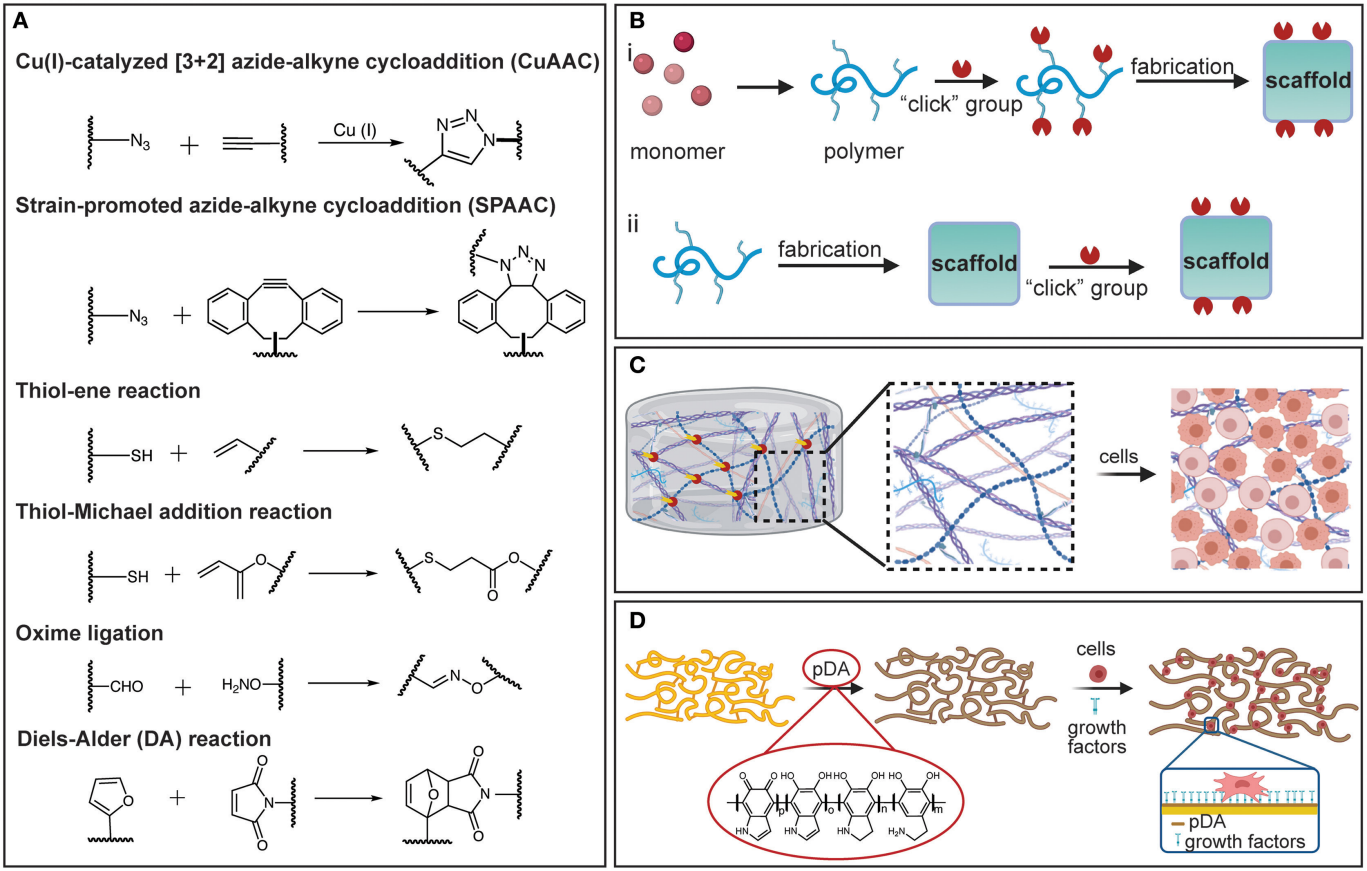
**(A)** Representation of various click chemistry reactions used in the fabrication of polymeric scaffolds. **(B)** Fabrication strategies based on click chemistry: (i) pre-click and (ii) post-click. **(C)** Schematic illustrating a hydrogel crosslinked by click chemistry and functionalized with ECM-based molecules to promote cell attachment and differentiation. **(D)** Schematic showing a PDA-coated electrospun scaffold decorated with growth factors to promote cell differentiation. [**(B–D)** were created with BioRender.com].

Click chemistry (Kolb et al., 2001; Zou et al., 2018) has emerged as a potent chemical modification strategy for scaffold biochemical functionalization, primarily because it demonstrates modularity, high reactivity, superb selectivity, high yield, and mild reaction conditions (Jiang et al., 2014). This approach offers many attractive possibilities for bioconjugation, accordingly enabling tailored and well-defined properties of polymeric scaffolds to be acquired via a wide range of surface and bulk functionalizations. Various click chemistry tactics, including copper(I)-catalyzed azide-alkyne cycloaddition (CuAAC) (Meldal and Tornøe, 2008), strain-promoted alkyne-azide cycloaddition (SPAAC) (Prescher et al., 2004; Laughlin et al., 2008), thiol-X reaction (Hoyle and Bowman, 2010; Daniele et al., 2014), Diels-Alder (DA) reaction (Nimmo et al., 2011; Tasdelen, 2011), and oxime ligation (Kalia and Raines, 2008), have been used to modify the chemistry of biomaterials (Xi et al., 2014; Zou et al., 2018) (Figure 2A). In the CuAAC chemistry reaction, a terminal alkyne reacts with an azide group to form a stable triazole ring. CuAAC chemistry has been used to attach functional ligands to the surface of PCL fibers (Lancuški et al., 2013); specifically, the PCL was end-functionalized with an azide moiety before electrospinning and, subsequently, bioactive groups containing alkyne moieties were conjugated at the fiber surface. The enhanced surface hydrophilicity imparted by the conjugated bioactive groups promoted cell attachment without reducing the molecular weight (Lancuški et al., 2012). Because of the toxicity of Cu(I) in biological systems, the SPAAC reaction has gained increased interest as a metal-free alternative approach, extending the usage of click chemistry in physiological settings. SPAAC has been used to functionalize the surfaces of fibers and develop *in situ* hydrogels with controlled crosslink densities (Zheng et al., 2012). Additionally, the functionalization and synthesis of polymeric materials by thiol-X reactions under mild conditions are characterized by simplicity and efficiency (Lowe, 2010). Among different thiol-based reactions, radical-mediated thiol-ene and thiol-yne are the most exploited in TE applications. However, because the former treatment uses UV light that may damage the cells and the tissue, the applicability of this chemical method is limited. In view of this obstacle, nucleophile-mediated thiol-X reactions, such as thiol-Michael addition, have been considered as plausible substitutes (Hoyle et al., 2010). In addition, oxime ligation is a reaction that can be safely implemented both *in vitro* and *in vivo*, because it can be performed at room temperature without the need of a metal catalyst and the application of UV light.

Click chemistry can be applied during the scaffold fabrication to modulate crosslink polymerization of functional monomers, or as a post-click functionalization strategy to impart the surfaces of polymeric scaffolds with specific functional groups to elicit cell attachment (Figure 2B). The high-efficiency reactions and excellent bond stability that characterize this method enable the crosslinking reactions to be precisely controlled to achieve tunable crosslink densities. Accurate tuning of the crosslink density is critical in the fabrication of hydrogel scaffolds. For instance, azide or alkyne terminal functionalities can be introduced in macromolecules, such as PEG and hyaluronic acid (HA), or in the presence of Cu(I) through the CuAAC crosslinking reaction, producing “clickable” hydrogels with tunable morphological and mechanical properties (Crescenzi et al., 2007; Gopinathan and Noh, 2018). Click chemistry also provides a facile way to introduce reactive and clickable sites directly on functional polymers. An example is the incorporation of free thiol groups in poly(3-mercaptopropyl) methylsiloxane-based fibers partially photo-crosslinked by UV illumination during electrospinning, followed by maleimide terminated poly(N-isopropyl acrylamide) brushes added to the fiber surface via a thiol-Michael addition reaction (Yang et al., 2012). The resulting fibers exhibit a thermosensitive behavior that may be desirable in several TE applications (Yang et al., 2012). In addition, because most synthetic polymers utilized in TE are lacking cell-binding moieties and often exhibit hydrophobic behavior, click chemistry can be used to decorate scaffolds with short amino-acid sequences that bind to the receptors on the cell surfaces and mediate cell attachment via specific surface-ligand biological signals. The most commonly immobilized peptide for surface and bulk modification is arginylglycylaspartic acid (RGD), for which the signaling domain is derived from fibronectin and laminin. In addition, several other peptide sequences, such as the Tyr–Ile–Gly–Ser–Arg (YIGSR) and the Ile-Lys-Val-Ala-Val (IKVAV), have also been immobilized on the surface of various biomaterials (Shin et al., 2003).

Although *in vivo* studies are critical for evaluating the performance of a scaffold, decoupling and elucidating all possible mechanisms that dictate cell fate is cumbersome because of the complex and sophisticated nature of most native biological environments. To disentangle the complexity of *in vivo* phenomena and elucidate how cells receive and transmit information, it is imperative to employ systems that can assay cell functions in well-defined settings that imitate native environments. To face this challenge, click chemistry has been used to functionalize specific scaffold sites and create 3D patterns for *in vitro* replicating and evaluating biological processes and environments (Wylie et al., 2011; Azagarsamy and Anseth, 2013; Truong et al., 2015). For instance, 3D-patterned hydrogels have been produced from biocompatible click reactions in conjunction with orthogonal photo-chemistries to sequentially introduce and remove biochemical and biophysical signals with precise spatiotemporal control (DeForest et al., 2009; DeForest and Anseth, 2011, 2012; Ruskowitz and DeForest, 2018). Enzymatically degradable hydrogel platforms with photo-patterned biomacromolecules introduced by sequential click reactions have enabled the detection and promotion of specific cellular functions and the direct observation of cellular processes, such as migration, proliferation, and morphology evolution (DeForest and Anseth, 2012). The SPAAC reaction has been used in hydrogel synthesis and the thiol-ene reaction to conjugate biomolecules (DeForest and Anseth, 2012). Thiol-ene reactions have also been used to introduce bioactive peptides in photo-degradable hydrogels, which were then photo-cleaved from the hydrogel network by UV radiation, enabling spatiotemporal control over peptide concentration (DeForest et al., 2009; DeForest and Tirrell, 2015). The foregoing studies have demonstrated that cell attachment, proliferation, and motility can be controlled, and cell behavior can be confined to patterned regions to provide platforms for studying how cells respond to chemical and mechanical cues (Figure 2C).

In addition to the click chemistry, which has been rapidly implemented in various biomaterial functionalizations, PDA treatment has also emerged as a facile and versatile chemical functionalization method (Madhurakkat Perikamana et al., 2015; Cheng et al., 2019; Malollari et al., 2019). The principal chemical modification strategies before the introduction of PDA were deposition of self-assembled monolayers (SAMs), plasma-assisted surface chemistry modification (PASCM), and layer-by-layer (LBL) deposition. However, SAMs require compatible chemistry between the surface and the absorbate, surface chemistry functionalization by PASCM is susceptible to alteration with time, and LBL deposition requires multiple coating cycles (Ryu et al., 2018). Since its introduction (Lee et al., 2007), PDA has been extensively used to functionalize a broad range of surfaces, including metals and ceramics, as well as materials exhibiting low surface energy. Depositing a conformal PDA coating to the surfaces of the latter materials not only improves the solubility and stability of the scaffold, but also provides multi-functionality. This is because the reactivity of the chemical residues in the PDA structure toward nucleophilic amines and thiols yields a superior capacity for immobilizing and conjugating a variety of molecules through Michael addition and Schiff base formations (Yang et al., 2014). DNA, drugs, cells, minerals, peptides, and proteins have been functionalized on PDA-modified substrates for diverse TE applications (Ryu et al., 2010; Tsai et al., 2014; Cheng et al., 2019; Li et al., 2020; Rühs et al., 2020). It has been reported that PDA can promote cell adhesion to various substrates (Ku and Park, 2010). In fact, the former study showed that glass, polydimethylsiloxane, silicone rubber, polytetrafluoroethylene, and polyethylene, which are highly resistant to cell attachment (adhesion), when coated with PDA demonstrate cell adhesion characteristics comparable to those of gelatin-coated substrates seeded with human umbilical cord vascular endothelial cells.

It is also known that PDA enhances cellular interaction with biomaterials by immobilizing cell adhesive ECM biomolecules. Specifically, PDA can serve as a primer for the subsequent conjugation of different cell adhesive moieties via covalent interactions between catechols and the amines or thiols of biomolecules (Figure 2D). Several PDA-coated polymer substrates can be functionalized with adhesive peptide sequences derived from ECM-based biomolecules, such as fibronectin, laminin, and growth factors (Cai et al., 2013). For example, it has been observed that PDA-coated poly(lactic acid-co-ε-caprolactone) immobilized dual bioactive factors, i.e., cell adhesive RGD-containing peptide and basic fibroblast growth factor (bFGF) for vascular grafting (Lee et al., 2012). The immobilization of RGD and bFGF influenced cell migration and proliferation, respectively, whereas the co-immobilization exhibited a synergistic effect, confirming that PDA can serve as a functionalization platform for vascular grafts. While PDA has been mostly used for post-functionalization, it is also of particular interest in hydrogel fabrication. For instance, PDA-crosslinked hydrogels, such as HA-catechol hydrogels, confer high stability, tunable mechanical properties, and minimal toxicity (Ryu et al., 2011; Hong et al., 2013). Although PDA has been proven to be a multifaceted chemical toolbox and PDA-coated scaffolds demonstrate enhanced adhesion, proliferation, and differentiation of various cells, the long-term *in vivo* toxicity and integrity of PDA during scaffold degradation require further in-depth investigation.

The above synopsis of surface and bulk chemical modification methods of the scaffold surfaces discloses a strong correlation between the scaffold chemistry and the host tissue response. The immunological response of the host tissue following scaffold implantation or injection can affect tissue regeneration. Consequently, a fundamental understanding of the effect of scaffold surface chemistry on cellular behavior is of utmost importance in the design of biomaterials that can modulate host immune reactions.

### Structure and Morphology

The materials and fabrication methods used in scaffold engineering must be versatile to allow tailoring of various key structure parameters (e.g., fiber diameter, porosity, and fiber alignment) and, thus, produce a scaffold that closely mimics the ECM of the tissue of interest. Variations of these parameters lead to different cellular responses (Jenkins and Little, 2019). One of the tunable parameters in fibrous scaffolds is the fiber diameter that has been proven to modulate cell differentiation (Christopherson et al., 2009; Wang et al., 2012; Bean and Tuan, 2015; Ghanian et al., 2015; Nguyen et al., 2016; Narayanan et al., 2020) and can be controlled by adjusting the fabrication process conditions (Elkasaby et al., 2018) and the degree of crosslinking (Shields et al., 2004; Gonçalves de Pinho et al., 2019). As an example, the alignment of 3-μm-diameter fibers was found to enhance the arrangement, growth, and differentiation of myoblasts, contrariwise to 300-nm-diameter aligned fibers (Narayanan et al., 2020). Changes in fiber diameter have also been observed to impact gene expression and phenotypic markers (Bashur et al., 2009; Noriega et al., 2012; Erisken et al., 2013; Lee et al., 2017). Similarly, fiber diameter has been shown to affect cell adhesion and proliferation (Erisken et al., 2013; Entekhabi et al., 2016). For instance, tendon fibroblast seeding on electrospun scaffolds consisting of poly(lactic-co-glycolic acid) (PLGA) nanofibers demonstrated an enhancement of cell proliferation, total collagen, and proteoglycan, whereas their microfibrous version demonstrated increased expression of phenotypic markers, such as collagen I, II, and V and tenomodulin (Erisken et al., 2013). These findings indicate that nanofibers resemble an injured ECM and stimulate regeneration, whereas microfibers aid to maintain the fibroblastic phenotype. The fiber diameter also exhibits a significant effect on cell infiltration, which is critical to 3D scaffold engineering. Indeed, it has been discovered that the larger the fiber diameter the better the infiltration of human venous myofibroblasts and more homogeneous the cell delivery in the scaffold (Balguid et al., 2009). Other studies have revealed a considerable effect of fiber diameter on the cell morphology and size (Bashur et al., 2009; Hsia et al., 2011; Daud et al., 2012; Kuppan et al., 2015; Lee et al., 2017) as well as on the cell migration velocity and distance range (Wang et al., 2010; Binder et al., 2013; Kievit et al., 2013; Meehan and Nain, 2014; Ottosson et al., 2017; Estabridis et al., 2018). However, fixing the fiber diameter for a particular cell behavior is not possible because it depends on the type, size, and shape of cells, the scaffold material, and the fabrication process, which affects the scaffold structure and fiber diameter (Noriega et al., 2012; Zouani et al., 2012).

Similar to the fiber diameter, microgroove patterns can influence the cell morphology and, in turn, the proliferation rate and spreading of the cells (Thakar et al., 2009) as well as cell differentiation (Watari et al., 2012; Abagnale et al., 2015). For instance, seeding of human mesenchymal stem cells (hMSCs) isolated from lipoaspirates and cultured in a differentiation medium on topographically patterned structures with grooves 5 μm in width and ridges of different widths promoted osteogenesis for 2-μm-wide ridges and adipogenesis (fat cell formation) for 15-μm-wide ridges, whereas 650-nm-wide ridges stimulated differentiation toward osteogenic and adipogenic lineages (Abagnale et al., 2015), in contrast with the findings of a previous study (Watari et al., 2012).

Another scaffold parameter that can be adjusted is the fiber alignment or the structure morphology, which have also been correlated with various cellular responses (Sundararaghavan et al., 2013). In particular, aligned fibers have been observed to enhance stem cell differentiation conversely to randomly oriented fibers (Christopherson et al., 2009; Tijore et al., 2015; Abarzúa-Illanes et al., 2017; Sperling et al., 2017). However, other findings suggest that random fibers represent a more suitable environment for stem cell differentiation (Lins et al., 2017). Enhanced cell proliferation has also been linked to fiber alignment, although there have been reports of increased cell affinity to attach on either aligned or randomly dispersed fibers (Lim et al., 2010; Ren et al., 2013; Lins et al., 2017). For instance, while increased keratocyte proliferation occurred on the aligned nanofibers of a gelatin/poly(L-lactic acid) (PLLA) scaffold, corneal epithelial cells exhibited a higher proliferation on randomly oriented fibers of the same material (Yan et al., 2012), revealing a unique cell response to scaffold fiber alignment. Moreover, correlations between cell shape and fiber orientation have been observed in several investigations. Typically, cells tend to elongate in the direction of fiber alignment and assume a spherical or polygonal shape when seeded on scaffolds with randomly oriented fibers (Bashur et al., 2009; Vimal et al., 2016). Surprisingly, while keratocytes spread randomly on fibers oriented in various directions and elongate along the direction of aligned fibers, corneal epithelial cells remain large, round, and flat on both randomly oriented and aligned fibrous gelatin/PLLA scaffolds (Yan et al., 2012). Additionally, the fact that cell migration occurs faster in the direction of fiber alignment (Ottosson et al., 2017) reveals a dependence of cell migration on both fiber diameter and alignment and highlights the importance of optimizing the scaffold morphology during the fabrication.

Pore scaffold architecture, including pore size, shape, and density, is of principal importance in cellular behavior. Generally, high porosity and large pores are associated with increased proliferation, differentiation, and gene expression (Lowery et al., 2010; Zhang et al., 2010; Zhang Q. et al., 2014; Nunes-Pereira et al., 2015; Di Luca et al., 2016c,d), although an opposite trend has been reported in some studies (Mandal and Kundu, 2009; Ng et al., 2018). For instance, in the case of silk fibroin porous membranes of fixed porosity fabricated by freeze-drying, large pores enhanced the proliferation of human dermal fibroblast (HDF) cells compared to membranes with small pores; however, membranes with high porosity and interconnectivity showed increased cell proliferation and cellular migration even for a much smaller pore size (Mandal and Kundu, 2009). These results suggest that high porosity and interconnectivity assist cell migration within the construct, facilitating cell growth in a compatible environment. Moreover, the pore size may exhibit a more profound effect on the proliferation of HDF cells compared to the fiber diameter and may also affect the cell morphology. For example, while 20 μm pores promoted cell elongation along the fibers of electrospun PCL scaffolds, 6.5 μm pores resulted in cell spreading across the fibers (Lowery et al., 2010). In general, pores larger than the cell stimulate cell alignment along a single fiber, whereas pores smaller than the cell favor cell bridging across fibers, resulting in slower movement and longer migration distances. This behavior may be attributed to the fact that large pores promote single-fiber cell attachment, which is conducive to faster cell migration. Nonetheless, the pore size effect on the cell behavior is strongly depended on the cell type and scaffold material (Nunes-Pereira et al., 2015).

Pore size and shape gradients are also important scaffold characteristics affecting cellular behavior. For example, *in vitro* experiments with hMSCs seeded on 3D printed PCL scaffolds with ~73% porosity and square or rhomboidal pores exhibited differentiation to chondrogenic and osteogenic cells, respectively (Di Luca et al., 2016b). Pore size gradients have also been linked to specific cellular differentiation and gene expression (Oh et al., 2007; Di Luca et al., 2016c,d), whereas radially varying porosity has been reported to yield cell behaviors characterized by gradient gene expression and cell differentiation and proliferation (Di Luca et al., 2016a). Through-thickness variation of the porosity and the fiber density accomplished by combining aligned and randomly oriented fibers in the scaffold structure and the incorporation of surface microwells have been correlated with increased cell migration and proliferation compared to controls of lower porosity consisting of randomly oriented fibers fabricated under the same conditions (Cheng et al., 2013; Pu and Komvopoulos, 2014; Pu et al., 2015).

Surface roughness is another scaffold parameter that strongly affects gene expression (Chen et al., 2017) and the adhesion (Milleret et al., 2012), morphology (Chen et al., 2017), differentiation (Faia-Torres et al., 2015), and proliferation (Ribeiro et al., 2015) of the cells. It has been found that rough and smooth surfaces prompt different cell responses. For instance, an investigation of the effect of surface roughness of electrospun poly(ethylene oxide terephthalate)/poly(butylene terephthalate) scaffolds on gene expression, differentiation, and morphology of hMSCs showed that relatively high surface roughness [i.e., root-mean-square (rms) roughness = 71 ± 11 nm] was beneficial to some osteogenic genes, whereas low roughness (i.e., rms roughness = 14.3 ± 2.5 nm) led to the expression of other osteogenic genes and a chondrogenic gene of the hMSCs, with the cells exhibiting a small spindle shape on the rough surfaces as opposed to an elongated and multipolar shape on the smother surfaces (Chen et al., 2017). In other studies, increasing the roughness of electrospun PLLA scaffolds decreased osteoblast proliferation but increased fibroblast proliferation (Ribeiro et al., 2015). The discrepancies about the effect of surface roughness on the cellular response, such as higher osteoblast proliferation and lower fibroblast proliferation on rough and smooth microporous poly(hydroxybutyric acid) membrane surfaces, respectively (Huaga et al., 2009), can be attributed to complex cell effects depending on the cell line and different chemical, topographical, and mechanical surface cues.

**Figure 3:**
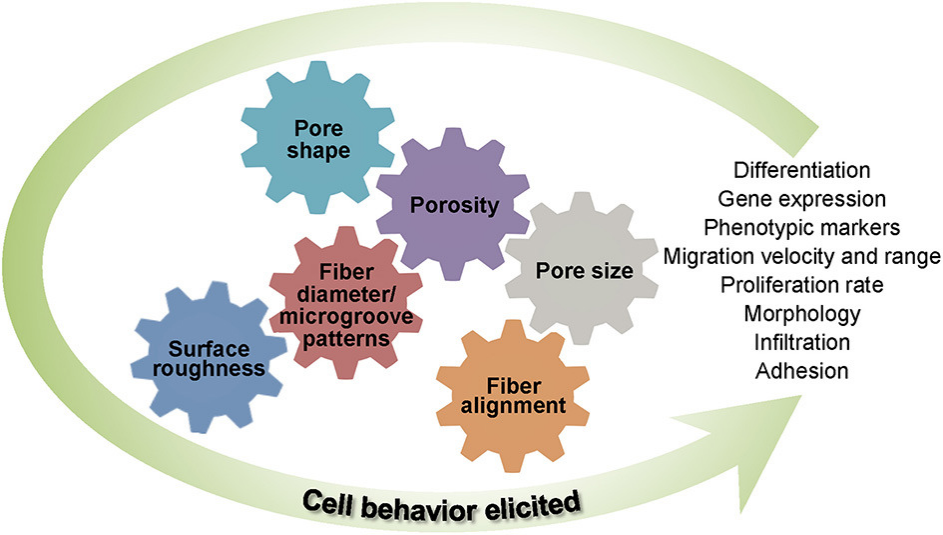
Important structure and morphology scaffold parameters affecting cell behavior.

All of the foregoing studies substantiate that the architecture, fiber diameter and alignment, pore size and shape, and surface roughness cannot be uniquely specified for all types of cells. Consequently, designing scaffolds to direct and promote specific cell behavior and morphology depends on the intrinsic specificities of the targeted application. Figure 3 provides a synopsis of the important structure and morphology parameters affecting cellular behavior in TE that were discussed in this section.

### Biodegradability

**Figure 4:**
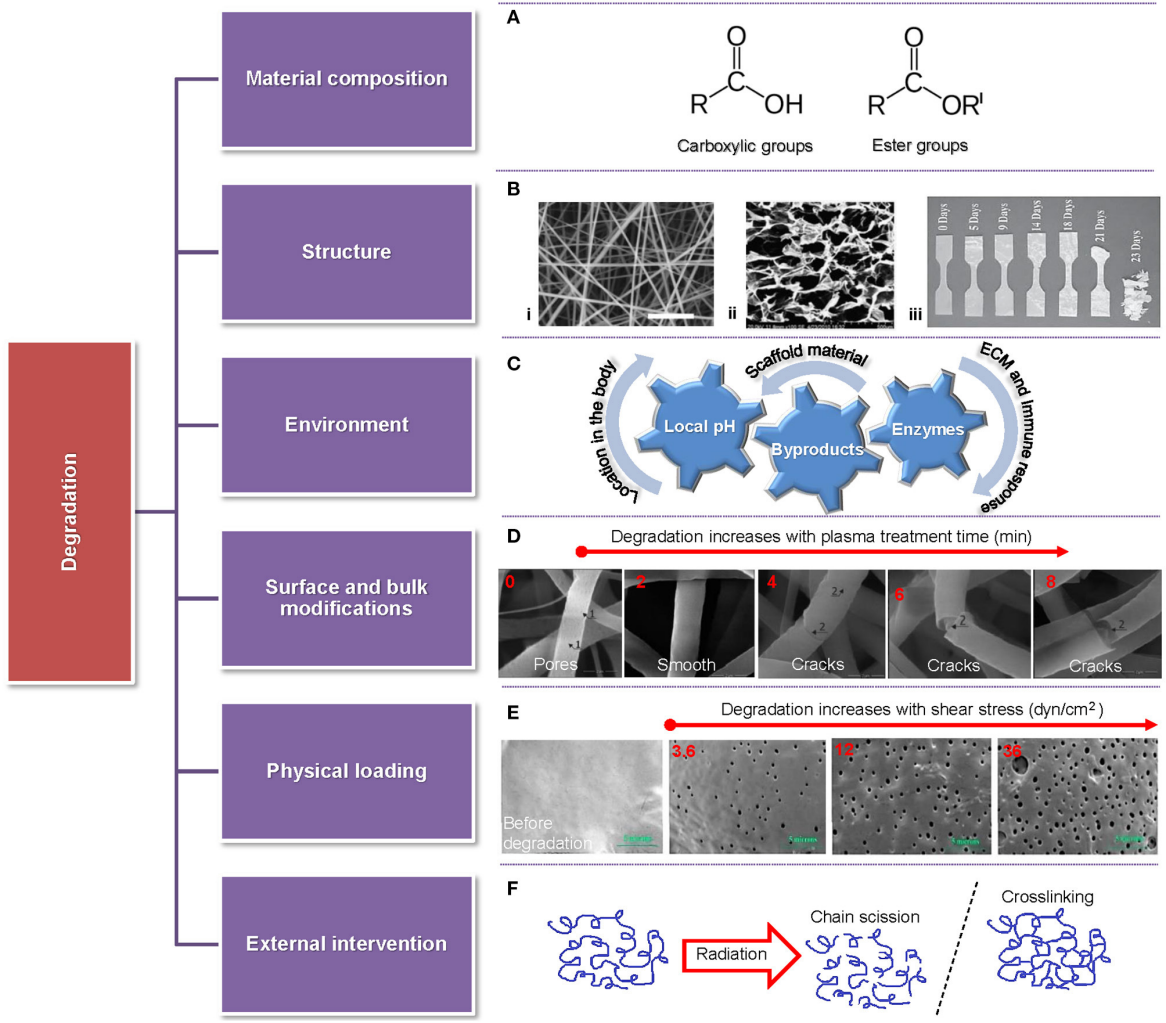
Overview of parameters affecting the degradation rate of scaffolds accompanied by representative examples. **(A)** Highly reactive functional groups attacked by water molecules. **(B)** Different scaffold structures: (i) fibrous (Pu and Komvopoulos, 2014), (ii) porous (Han et al., 2014), and (iii) solid (Weir et al., 2004). **(C)** Various environmental factors (local pH, byproducts, and enzymes) affecting scaffold degradation. **(D)** Fiber morphology vs. time of plasma treatment showing fiber degradation after a short treatment time and fiber cracking and breakage after long treatment time (Bolbasov et al., 2018). **(E)** Comparison between a fresh sample and samples degraded for 28 days in PBS at 32°C for shear stress increasing from 3.6 dyn/cm^2^ (left) to 36 dyn/cm^2^ (right) (Zheng et al., 2017). **(F)** Schematic of chain scission and crosslinking induced by radiation.

Biodegradability is of particular importance in scaffold engineering because it must be coordinated with tissue generation for the construct to maintain sufficient strength and effectively sustain the mechanical stresses in the tissue environment. Importantly, the byproducts of polymer biodegradation must be non-toxic to avoid an immune response and also extractable through normal body function. The degradation rate of biopolymer scaffolds is therefore of utmost significance because it is intertwined with the tissue viability. Specifically, if scaffold degradation is faster than wound healing and native tissue regeneration, the cells will be deprived of the ECM-like structure, the formed tissue can be defective or unfeasible, and the produced byproducts may not be promptly expelled from the body (Cajori et al., 1924; Pucino et al., 2019). Alternatively, too slow scaffold degradation may lead to scaffold encapsulation, triggering an immune response and poor integration or rejection from the host tissue (Balguid et al., 2009; Sanz-Herrera et al., 2009; Park et al., 2010; Holloway et al., 2014). The degradation rate is controlled by the material composition (functional groups), the scaffold environment (pH and enzymes) and structure (porous, hydrogels, and bulk), the surface and bulk chemical modification of the scaffold, the mechanical environment (physical loading), and the type of external intervention (e.g., ultrasound, heat, radiation, etc.), as illustrated in Figure 4. The degradation rate is typically quantified as a mass loss, despite the fact that not only affects the mass but also the crystallinity, geometry (shape), and topology of the scaffold (Suuronen et al., 1998; Annor et al., 2012; Zhang H. et al., 2014; Milošev et al., 2017). Other factors affecting the rate of scaffold degradation include the implant location and the patient's age, gender, and comorbidities (Roman et al., 2017).

The degradation rate of polymeric scaffolds strongly correlates with the material composition, the polymer molecular architecture (e.g., side groups, aromatic groups, double or triple bonds, and crosslinking), and the fabrication method (e.g., blending and copolymerization), which controls the degree of chain scission (Liu et al., 2012; Ferrari et al., 2017; Ferreira et al., 2019; Keirouz et al., 2020; Sadeghi-avalshahr et al., 2020) and modulates the biodegradability, hydrophilicity, and biocompatibility as well as cell adhesion, proliferation, growth, and antibacterial activity in TE applications (Gao et al., 2019). Polymer crosslinking reduces the degradation rate (Bi et al., 2011; Kishan et al., 2015; Chen et al., 2020), whereas the incorporation of a high fraction of nanoparticles (NPs) to play the role of bioactive sites in the scaffold may increase the degradation rate (Mehrasa et al., 2016; Radwan-Pragłowska et al., 2020), although some studies have shown an opposite effect (Mehrasa et al., 2016; Park et al., 2018). The degradation rate can also be tuned by chemical modification methods or by adding NPs that can neutralize acidic products (Zhang H. et al., 2014; Shuai et al., 2019).

Biopolymer degradation is a manifestation of several distinct but non-mutually exclusive mechanisms, namely hydrolytic degradation (chain scission by water molecules), enzymatic degradation (enzyme catalysis), oxidative degradation (radicals produced from peroxides), and physical degradation associated with various factors, such as loading, wear, and swelling (Brannigan and Dove, 2017). The most common synthetic polymers used in TE, i.e., polyhydroxy esters, such as PLLA, PCL, PGA, PEO, and PVA, degrade abundantly by hydrolytic chain scission, where the loss of monomers and oligomers activates bulk or surface erosion mechanisms, depending on the dynamics of diffusion-reaction phenomena (Drury and Mooney, 2003; You et al., 2005; Annor et al., 2012), and generates acidic byproducts (Idaszek et al., 2013; Kianpour et al., 2020). Alternatively, because the macromolecules of natural polymers, such as collagen, HA, alginate, and chitosan, are similar to the native ECM, they are prone to enzymatic degradation (Lam et al., 2009; Annor et al., 2012). Particularly, collagen is naturally degraded by collagenase and proteases, allowing for locally controlled degradation by the cells in the tissue (Drury and Mooney, 2003; Annor et al., 2012). Accordingly, to tailor the degradation rate, natural polymers have been used in conjunction with synthetic polymers (Wan et al., 2008; Fu et al., 2014).

Degradation also changes intrinsic topography features of the scaffold, such as fiber roughness and diameter, porosity, and wettability, depending on the surface area-to-volume ratio of the scaffold, consequently affecting the cellular behavior (Lam et al., 2009). A larger surface area-to-volume ratio typically leads to faster degradation. Indeed, a comparison between electrospun biodegradable L-tyrosine-based PU membranes and thin films of the same material revealed a higher degradation rate for the electrospun membranes than the films, with the hydrolytic degradation rate of the membranes showing a dependence on blending ratio (Spagnuolo and Liu, 2012). The surface area-to-volume ratio is linked to the wetting angle (hydrophilicity/hydrophobicity) and water intake (swelling) of the scaffold. For instance, electrospun poly(D, L-lactic acid) (PDLLA) and PEO/PLA (PELA) block copolymer scaffolds have been reported to demonstrate faster degradation compared to casted film counterparts, especially in the early stage of the hydrolysis process, with the difference in degradation rate between the two materials decreasing after 10 weeks of incubation in phosphate buffered saline (PBS) at 37°C. It was also reported that the fibrous PDLLA exhibited a larger wetting contact angle than the fibrous PELA and even larger than the cast PDLLA (Cui et al., 2008), in agreement with the findings of an earlier investigation (Cui et al., 2006).

Loading effects on scaffold degradation have received relatively less research attention. Degradation studies of 3D printed PLGA in a PBS solution have shown faster scaffold degradation in a shaking incubator than in a microchannel with recirculating solution or static incubators, with the degradation rate of the samples in the static incubator being between those of the other two cases, illuminating the importance of the effects of fluid flow and loading on the degradation of PLGA scaffolds (Ma et al., 2018). Similarly, cyclic loading has been observed to accelerate PLGA degradation compared to static loading (Yang Y. et al., 2010), whereas high fluid shear rates in drug-releasing PLGA films used in vascular drug-eluting stent applications have been correlated to a faster release of sirolimus particles from the films, matching the degradation rate of the PLGA matrix (Zheng et al., 2017).

Various external factors (e.g., ultrasound, heat, and radiation) encountered during scaffold sterilization, patient therapy, and scaffold surface modification can also influence the rate of scaffold degradation by indirectly affecting the aging, crosslinking, morphology, hydrophilicity, and physical, chemical, and mechanical properties of the polymer material in combination with the cellular behavior (Yixiang et al., 2008; Rediguieri et al., 2016). For instance, under typical cancer therapy radiation levels, PCL undergoes backbone changes that have been associated with crosslinking and faster degradation (Cooke and Whittington, 2016). *In vivo* studies of electrospun PLLA scaffolds coated with titanium by reactive magnetron sputtering in nitrogen plasma atmosphere have shown that plasma treatment can mitigate inflammatory response, increase wettability, and stimulate cell attachment to the scaffolds, while, at the same time, expediting the commencement of scaffold degradation (Bolbasov et al., 2018).

The degradation byproducts may modify the local pH of the media surrounding the cells, consequently affecting cell energy metabolism, matrix synthesis, and cell behavior (Jones et al., 2015). An acidic pH environment may promote fibroblast proliferation and the migration and regulation of bacterial colonization, with keratinocytes showing optimal migration at more alkaline pH levels, e.g., pH ≈ 8.5 (Sharpe et al., 2009). Furthermore, enzyme activity is pH sensitive. For instance, protease exhibits a peak activity in the pH range of 7–8 and a decreased activity in acidic media, suggesting the need for a balanced pH for this enzyme because an excess of proteases hinders the wound healing process (Greener et al., 2005). Investigations of the pH effect on the metabolism of chondrocytes embedded in agarose gel have shown that even for a narrow pH range of 6.6–7.3 acidic pH suppresses the production of lactate and increases glycosaminoglycan synthesis without affecting the synthesis of collagen, suggesting that metabolic activities and the biosynthetic ability of chondrocytes are strongly influenced by the media pH (Wu et al., 2007). As mentioned earlier, many synthetic polymers generate acidic byproducts upon degradation, consequently stimulating local inflammation and interfering with the healing process. Therefore, other biopolymers, such as biodegradable PU foams and polycarbonate, which exhibit good biocompatibility, reduced inflammatory response, and controlled degradation to non-cytotoxic byproducts, have been used to promote cell attachment, collagen deposition, and keratinocyte growth and retention, especially for skin TE applications (Hafeman et al., 2011; Greenwood and Wagstaff, 2016; Xu and Guan, 2016; Brannigan and Dove, 2017).

Several studies have been focused on the effect of the scaffold degradation rate on cellular behavior, including adhesion, proliferation, differentiation, and metabolism. For example, the slow degradation of electrospun PCL scaffolds maintained the rigidity needed for myofibroblast differentiation and the secretion of higher levels of matrix components, whereas the decreased rigidity of the faster degrading poly(glycolic acid)-poly(4-hydroxybutyrate) scaffolds led to the accumulation of acidic byproducts, reduced matrix production, poor cell proliferation and differentiation, and undesirable collagen crosslinking (Balguid et al., 2009). Porous silk fibroin scaffolds prepared with the salt leaching process and two different solvents (i.e., hexafluoroisopropanol and aqueous solution) demonstrated remarkable differences in degradation rate exclusively due to the process condition (solvent), conversely to chemical modification followed by *in vitro* seeding with human bone marrow stem cells in an osteogenic medium for 56 days, where the faster degrading aqueous-derived scaffold exhibited a higher metabolic rate (i.e., higher glucose consumption and lactate production) during osteogenesis due to the enhancement of proliferation and osteoblastic differentiation (Park et al., 2010). In another study, 3D printed porous PLGA scaffolds showed similar degradation rates with that of new bone generation and maturation after 24 weeks of implantation in a rabbit model (Ge et al., 2009). The degradation of injectable porous hydrogels is another emerging application area. This is because TE relies on the scaffold's capacity to effectively release drugs, proteins, and nanoparticles to aid the wound healing process. An *in vitro* study of two porous injectable hydrogels, namely serum bovine albumin (BSA) and vascular K_2_(SL)_6_K_2_ polypeptide/BSA (KK-BSA) crosslinked via sulfydryl groups by Ag^+^ ions, showed that KK-BSA hydrogel degraded faster than the BSA and completely within 7 days of soaking in PBS, suggesting that antibacterial units (Ag^+^) and vascular polypeptide can be released in the early stage of wound healing, matching the physiological drive for wound healing (Cheng et al., 2020).

### Mechanical Behavior

The cells sense the physicochemical characteristics of their microenvironment via surface mechanoreceptors and accordingly respond by actively remodeling it. Although various factors of a cellular microenvironment (e.g., cell interactions, growth factors, and ECM biochemistry and structure) contribute to vital signaling for the cells, the signals generated by the ECM play a particularly crucial role in the biochemical response of the cells (Dvir et al., 2011). The ECM influences the basic biological functions and the fate of the cells through both biochemical interactions (e.g., growth factors and adhesive motifs) and mechanical cues (e.g., stiffness and deformability). Mechanical signals have profound effects on cell differentiation, proliferation, and death, greatly affecting tissue growth *in vivo* (Vogel and Sheetz, 2006; Wang et al., 2009). In the design of scaffolds for TE it is often required to recapitulate the modulus and ductility of a range of native environments. However, the complexity of the cellular environment requires an awareness of its physical, biochemical, and mechanical properties for the successful fabrication of functional tissue analogs.

One of the main aspects affecting cell micromechanics is the scaffold structure characteristics, such as porosity, pore size, fiber diameter, and fiber alignment (Kennedy et al., 2017). For fibrous constructs, such as electrospun scaffolds, altering the fiber diameter influences both the cell behavior and the cell morphology, such as the cytoskeletal arrangement and adhesion sites, ultimately changing the intracellular tension forces that direct cell phenotypes (Badami et al., 2006; Noriega et al., 2012). Changes in the fiber diameter can greatly affect the fiber stiffness. Indeed, because large-diameter PCL and PVA fibers are more amorphous due to the fact that they possess a less aligned molecular structure, they exhibit a lower elastic modulus compared to small-diameter PCL and PVA fibers that show increased crystallinity and, accordingly, higher elastic modulus (Wong et al., 2008; Stachewicz et al., 2012). It is well-accepted that cell-matrix interactions depend on the degree of fiber alignment (Baji et al., 2010; Wen et al., 2014). Fiber alignment in scaffolds that mimic the ECM of the tissue, such as blood vessels (Ma et al., 2005) and nerve tissue (He et al., 2010), have been found to direct cell phenotypes. Even though the fiber alignment does not directly impact the elastic modulus of the fibers, it influences the stiffness experienced by the cells. Because the fibers display a higher effective stiffness in the longitudinal direction than the transverse direction, cell attachment and migration mostly occur along the axial direction of the fibers. Moreover, the packing density of the fibers may also influence the effective stiffness. For instance, bundling of collagen fibers increases the effective stiffness (from ~1 kPa for a single fiber to ~5 kPa for fiber bundles), enhancing the cell adhesion and migration rate (Doyle et al., 2015).

While changing key structure parameters of scaffolds, such as the pore size, fiber diameter, and fiber alignment, is an effective method for tuning the mechanical properties of scaffolds, the material chemistry can also be used to independently modify the mechanical behavior of the scaffolds. Thus, the mechanical properties of polymeric scaffolds can be altered by changing the photopolymerization time or the number of photoreactive groups (Kennedy et al., 2017). These approaches can be employed during the formation of hydrogels or electrospun scaffolds to control the mechanics while maintaining the structure architecture. For example, a PEG dimethacrylate nanofiber hydrogel matrix with tunable elasticity fabricated by integrating electrospinning with photopolymerization was used to study the effects of scaffold elasticity on the differentiation rate of hMSCs to vascular cells *in vitro* (Wingate et al., 2012). An increase in polymerization time enhanced the degree of crosslinking, producing scaffolds with elastic modulus in the range of 2–15 kPa, consistent with *in vivo* stiffness. It was also found that the matrix elasticity instigated the cells to express different vascular phenotypes that demonstrated high differentiation efficiency, with softer scaffolds resulting in upregulation of the endothelial cell markers (Flk-1) and stiffer scaffolds eliciting smooth muscle cell (SMC) markers. The former study (Wingate et al., 2012) also illuminated the importance and capacity of local elasticity to control MSC differentiation to endothelial or SMC-like cells, leading to a vascular tissue regeneration in which the endothelial cell layer was softer than the SMC layer. In another investigation, electrospun photoactivatable methacrylated HA scaffolds induced chondrogenic differentiation of hMSCs by altering the fiber mechanics through the crosslink density and the cell adhesiveness mainly through the concentration of conjugated tripeptide RGD motifs (Khetan et al., 2013). It was also shown that fiber mechanics govern gene expression, with softer fibers promoting the expression of chondrogenic markers and the adhesive motifs at the fiber surface directing cell adhesion, proliferation, and migration. Studies dealing with the effects of the matrix stiffness and spatial distribution of cell-adhesive motifs on the attachment and differentiation of stem cells have demonstrated that both the matrix stiffness and the presence of adhesive cues can override other physical effects affecting the stem cell fate (Ye et al., 2015; Yevick et al., 2015). The scaffold mechanics can also be adjusted by varying the weight ratio of polymer blends (Vatankhah et al., 2014), although this may alter the surface roughness and hydrophilicity of the scaffold and, consequently, change the cell behavior.

Likewise, both the strength and the stiffness of bulk hydrogel scaffolds depend on the rigidity of the polymer chains and the crosslink density. Sequential photopolymerization allows for spatiotemporal control of the hydrogel stiffness (Leijten et al., 2017). The degree of photopolymerization, which depends on the irradiation dose and the crosslink density, can be tuned to produce unreacted functional groups in the matrix, which can enhance the hydrogel's mechanical properties through a second crosslinking reaction at a certain time (Guvendiren and Burdick, 2012; Guvendiren et al., 2014). In a specific case, matrix metalloproteinase (MMP)-degradable PEG hydrogels of different stiffness were used to examine the effect of the matrix modulus on the behavior of valve interstitial cell myofibroblasts seeded in 3D scaffolds as compared to flat hydrogel counterparts (Mabry et al., 2015). It was found that *in situ* stiffening of cell-laden PEG hydrogels by thiol-ene chemistry is an effective means of independently varying the scaffold mechanics at different length scales. The former study also provided insight into how cells sense mechanical signals originating from multiple length scales. While chemistry can be a powerful approach when modulating the micromechanical characteristics of scaffolds, modifying the mechanics by either blending or photopolymerization introduces a new surface chemistry, which may affect the cell behavior, the cell affinity to attach onto the scaffold surface, and the interaction of the cells with their microenvironment.

Although scaffolds undergo dynamic mechanical changes due to degradation and loading effects, the cells can also change the mechanical properties of scaffolds through the deposition of ECM, the application of cellular tractions, or even the reconstruction of the surrounding tissue. A scaffold implanted in the body experiences physiological loads (e.g., shearing by body fluids) that depend on the host tissue and/or cellular traction forces, which can induce deformations in the surrounding environment. Cellular traction forces manifest themselves during cell attachment. Cell seeding can result in scaffold contraction when there is a scaffold-tissue stiffness mismatch and the scaffold elasticity is inadequate to absorb the forces exerted by the moving cells. However, a small degree of local contraction is needed to assist cell migration and differentiation (Ulrich et al., 2010). In a study with a new dextran methacrylate hydrogel system that resembled the fibrous architecture of native ECM where the fiber stiffness, diameter, and alignment were controlled, lower fiber stiffness caused the cells to recruit neighboring fibers, effectively increasing the adhesive ligand density and enhancing cell adhesion and spreading (Baker et al., 2015). It was also shown that increasing the stiffness of the 3D fibrous constructs suppressed cell spreading and proliferation, whereas increasing the stiffness of flat hydrogel counterparts was conducive to MSC spreading and proliferation. The mechanical properties of scaffolds can also be affected by hydrolytic and/or enzymatic scaffold degradation, with the degradation rate exhibiting material dependence. For instance, PCL is a slowly degrading polymer that preserves its bulk mechanical properties for an extended period (~65 days), as opposed to PCL-PLGA blends that display significant deterioration of the mechanical properties and considerable mass loss (Baker et al., 2009).

**Figure 5:**
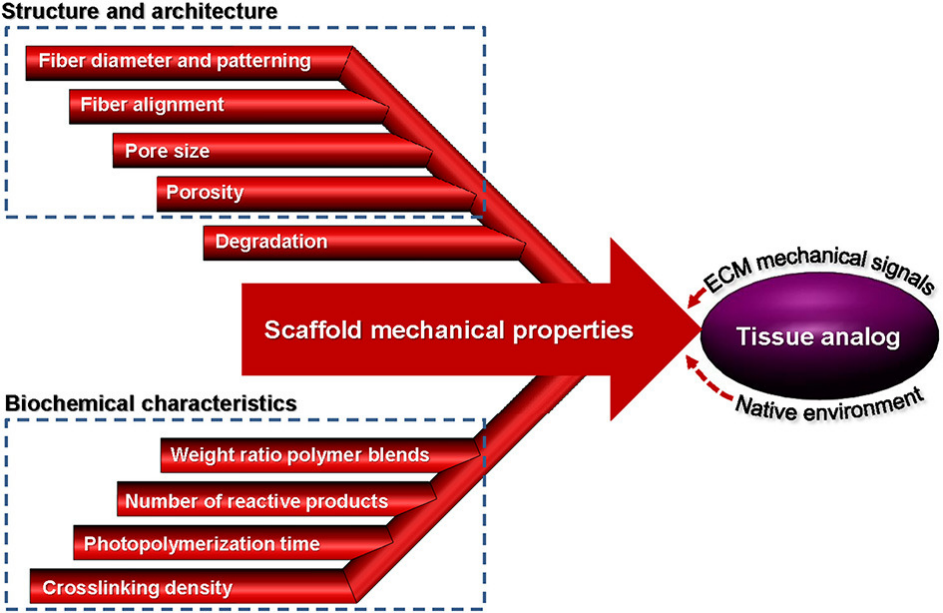
Parameters affecting the mechanical properties of scaffolds from fabrication to implantation used to create a tissue analog.

It is well-known that cells are dynamic systems responding to changes of their microenvironment by altering their morphology and applying tractions or by modifying their microenvironment through cell-mediated enzymatic degradation. However, more studies are needed to better understand the interplay between local degradation and cellular tractions and how the latter can influence differentiation and other cellular behaviors. Nevertheless, most studies conducted so far have established that the mechanical properties of synthetic scaffolds can dictate the cell fate and regulate the cell response. Figure 5 summarizes the parameters that play an important role in the mechanical behavior of scaffolds discussed in this section, including the cell-scaffold interface.

## Immunomodulation

Biomaterials for TE aim to replace damaged or diseased tissues with functional site-specific tissues. Although biomaterials properties, such as strength, porosity, degradability, and chemistry, are considered to be the main design principles for tuning the response of seeded cells and mimicking the nature of the native tissue, another critical factor for achieving successful clinical outcomes is the host tissue response to the implanted biomaterial. The immune system plays a vital role in host defense against pathogens, foreign bodies, and the tissue healing response following injury (Dellacherie et al., 2019). Since biomaterials are essentially foreign bodies, the immune-mediated tissue reaction to the presence of the foreign body is of utmost importance for the success of the TE strategy. Historically, in TE and regenerative medicine fields, immune responses were considered as challenges that had to be overcome because immunological reactions usually lead to restricted integration and regeneration of transplanted cells, tissues, and organs. However, in recent years, the design of biomaterials has shifted from suppressing the immune response to actively modulating it to promote synergy with the host environment (Sadtler et al., 2019; Stabler et al., 2019). Therefore, immunomodulatory biomaterials which promote or facilitate desired activation state or phenotype within the host immune cells have acquired widespread attention in the development of more effective biomaterials (Andorko and Jewell, 2017; Dziki and Badylak, 2018). To address the importance of immunomodulation in biomaterials design for TE applications, a brief overview of the role of the immune system in tissue repair and the role of physicochemical properties of scaffolds in directing immune responses is provided next.

**Figure 6:**
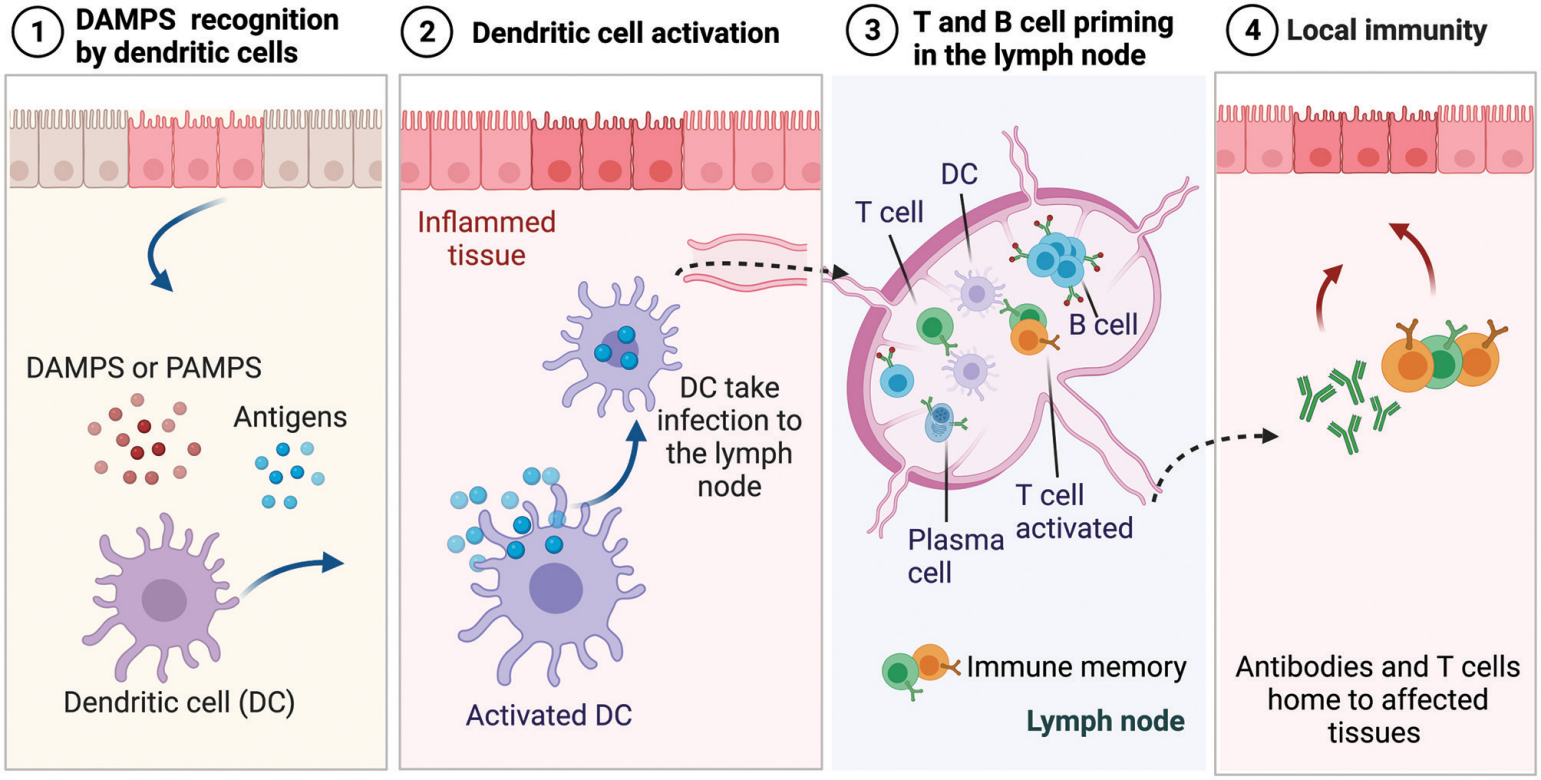
Overview of innate immune response upon injury or infection.

The immune system continually surveils the body to detect the invasion of harmful pathogens or tissue damage, and upon recognition of a threat, it activates an orchestrated inflammatory cascade of events (Medzhitov, 2008). The recognition of pathogens occurs through the release of molecules by dying cells that are uncommon and not typically found in the body. These molecules are referred to as danger-associated molecular patterns (DAMPS) and pathogen-associated molecular patterns (PAMPS) when occurring from various species of bacteria, viruses, and parasites (Iwasaki and Medzhitov, 2010), as shown schematically in Figure 6. During the innate immune response (i.e., the segment that provides rapid but less specific reaction) these patterns are recognized by tissue-resident immune cells that trigger the secretion of inflammatory signals, which are picked up by antigen-presenting cells, such as dendritic cells (DCs) and macrophages. Initially, neutrophils infiltrate the injury site and act non-specifically to clear pathogens associated with the injury, while secreting cytokines that recruit other immune cells, such as macrophages that clear up the cellular debris (Nathan, 2006; Selders et al., 2017). When the macrophages arrive at the injury site, they exhibit inflammatory M1 phenotype and clear up the cellular debris and the phagocytose pathogens. Once the threat is eliminated, phagocytes transition to anti-inflammatory phenotype M2, promoting tissue repair and generation of new blood vessels. While neutrophils and phagocytes comprise the first line of defense, they act with non-specificity because they cannot identify the pathogen they encounter. As described previously, during infection or acute injury during biomaterial implantation, pathogens or dying cells release antigens and threat signals. In the innate immune response phase, DCs, which are antigen-presenting cells, pick up the pathogen-derived antigens and threat signals and present them as surface activation signals on their membrane, including signals that induce their maturation and migration to the lymph nodes as well as major histocompatibility complexes responsible for presenting antigens to immature B and T cells (Dellacherie et al., 2019), as shown in Figure 6. Antigens and threat signals can also drain to the lymph nodes, where they bind and activate B cells to produce antibodies and/or activate resident antigen-presenting cells to prime T cells. Priming of immature T and B cells leads to the development of effector T cells and plasma cells and the development of memory T and B cells that can be activated when the same antigen is encountered (Kurosaki et al., 2015). The effector T cells and antibodies home to the affected tissue and participate in the adaptive immune effector phase.

Scaffolds for TE applications incorporate signals or cells which, upon implantation or injection, induce proliferation of the encapsulated cells, alter the phenotype of the infiltrating cells, and promote changes in tissue growth and function. Similarly, altering the physical properties of biomaterials can affect the immune response. Alteration in size and shape, composition, charge, and topography of biomaterial constructs can impact the intrinsic immunogenicity. For instance, physically confining the macrophage shape by micropatterning (i.e., elongated or pancake-like shapes) can affect the phenotype without the need to add exogenous cytokines (McWhorter et al., 2013). Also, the pore size of scaffolds can impact fibrotic capsule formation and shift macrophage phenotype. Studies have revealed a correlation between increased pore size of electrospun scaffolds and a shift toward M2 macrophage phenotype (Garg et al., 2013; Sussman et al., 2014). However, while porosity can be tuned to promote a regenerative environment by modulating macrophage phenotype, it may negatively impact the mechanical strength of the construct, which may be detrimental for recapitulating the strength of the native tissue. Changes in stiffness can also modulate host immune interactions, impacting both the biomaterial mechanics and its degradation rate. Modifying the extent of crosslinking and, in turn, the biomaterial stiffness can affect the M2-to-M1 macrophage ratio while, at the same time, altering the type of crosslinking; consequently, degradability can also influence immune cell recruitment and inflammatory response. For instance, changing the crosslinking of collagen scaffolds from glutaraldehyde to hexamethylene diisocyanate resulted in a 10-fold enhancement of neutrophil recruitment even after 28 days of subcutaneous implantation (Ye et al., 2010). Additionally, tuning the chemical properties of scaffolds can have a dramatic effect on the host response. Crosslinking agents, including carbodiimide or glutaraldehyde, which aim to strengthen ECM-based scaffolds, have been shown to promote an early pro-inflammatory immune cell phenotype and inhibit the degradation of ECM by macrophages (Valentin et al., 2009; Brown et al., 2012; Sadtler et al., 2016). Furthermore, the size and geometry of scaffolds affect responding immune cell phenotypes by altering foreign body reaction and fibrosis. Tuning the geometry of implanted materials can influence their host recognition and propagation of foreign body reactions. For instance, it has been shown that increasing the implant size is insufficient in battling foreign body responses and that spherically-shaped implants are essential for resisting host fibrosis (Veiseh et al., 2015). Also, the degradation products of ECM-based hydrogels have been reported to have a diverse effect on macrophage phenotype compared to particulate powder ones (Dziki et al., 2017; Sadtler et al., 2017).

The design of biomaterials which can actively modulate the immune response rather than circumvent or suppress it is of paramount importance to achieving tissue repair and regeneration. Leveraging the immunomodulatory capabilities of biomaterials requires control over their physicochemical features, careful design and selection of their morphology and architecture, and thorough *in vitro* and *in vivo* studies to reveal the interplay between the biomaterial and the resulting immune response.

## Recent Progress in Scaffolds for Skin and Brain Tissue Engineering

The previous sections were devoted to the main characteristics of scaffolds and an analysis of how the scaffold properties can be tuned to meet specific tissue characteristics, such as cellular behavior, mechanical integrity, biodegradability, and chemical cues recognition. This section provides an appraisal of the latest progress in two soft tissue applications and the results obtained through the implementation of the previously discussed design criteria integrated with biomaterials selection and delivery of small molecules and macromolecules (i.e., drugs, growth factors, etc.) to create functional scaffolds that elicit specific cellular responses. While there are many soft tissue applications, this section is focused on skin and brain tissues for several reasons. First, these two tissue types can be juxtaposed by considering their accessibility since the skin is significantly easier to access than the brain. Second, these tissues possess different levels of research maturity. Skin replacements have been engineered for decades with relatively good success, whereas brain tissue repair has not been studied as broadly as the skin, although there are still major challenges to be overcome before fully functional skin tissue can be created, mainly because of its highly heterogeneous and multilayered structure. Third, recent technological advancements, including but not limited to tissue/material interfaces and wearable electronics, demand more robust solutions (i.e., size scale and full thickness function) for both the brain and the skin. To recognize the fundamental properties that the skin and brain scaffolds must possess, it is informative to briefly describe the basic anatomy, biological function, and etiology of the respective native tissues before delving into the recent advancements in these TE applications. Specifically, the research progress reported here is categorized by the biomaterial of choice, while highlighting how the addition of drugs, growth factors, and particles can elicit the desired cellular behavior.

### Skin Scaffolds

The skin is the largest organ of the human body accounting for about 15–20% of the total body mass and the first line of the body's defense against the outside environment, microorganisms, UV radiation, and harmful chemical, biological, and physical effects. In addition, it plays a critical role in thermoregulation, endocrine regulation, exocrine secretion, and sensation (Hansen, 2009). The skin consists of three main layers, namely the epidermis, dermis, and hypodermis. The epidermis is the external layer and mostly consists of keratinocytes as well as melanocytes, Langerhans cells, and Merkel cells. It does not contain any blood vessels or lymphatics but has a few nerve terminals and its thickness depends on the body location, varying from ~0.05 mm in the eyelids to ~1.55 mm in the hand palms and the feet soles. Alternatively, the dermis is a very dense matrix mostly consisting of fibroblast cells that produce collagen and ECM constituents such as elastin, and its 3D random fibrous structure provides the skin with the needed strength and toughness. The dermis also contains hair follicles (HF), nerves, blood vessels, and sweat and sebaceous glands. The hypodermis is located below the dermis and contains adipocytes cells that store fat for energy-related processes (thermal regulation) (Moore et al., 2013; Stojic et al., 2019).

The wound healing process comprises several stages of hemostasis, including inflammation, cell proliferation and migration, angiogenesis, re-epithelization, appropriate synthesis, crosslinking and alignment of collagen, and ECM remodeling, which occur in cascade fashion, overlapping each other in an auto-regulated manner. During the hemostasis, blood vessels constrict and fibrin clots form, releasing pro-inflammatory cytokines and growth factors. The inflammatory phase is instigated when inflammatory cells migrate to the wound's ECM, and neutrophils, macrophages, and lymphocytes infiltrate the wound tissue to clean it. Macrophages lead to a transition from inflammation to proliferation that results in epithelial cell proliferation and migration, while fibroblasts and endothelial cells work synergistically to provide capillary growth, collagen, and granulation tissue. Finally, the remodeling phase occurs during which the density of vascular capillaries increases, the ECM approaches initial normal tissue, and physical contraction of the wound is instigated until full closure. All of the cells that participated in the healing process and no longer are needed undergo programmed apoptosis. Local factors that disrupt this process causing a delay or impairment of the wound healing process include oxygenation, infection, foreign body, and venous sufficiency. These local factors can be affected by systemic factors, such as age and gender, hormones, stress, ischemia, and other comorbidities. Local and systemic factors are non-mutually exclusive, both contributing to the healing process (Guo and DiPietro, 2010; Sorg et al., 2017) and the wide range of physical properties (Edwards and Marks, 1995).

Most of the needs in skin TE are for full-thickness skin wounds for which the body is incapable to fully regenerate the tissue through the normal healing process. These wounds may be due to burns, injuries, or disease (e.g., ulcerations due to cancer, diabetes, etc.). Third-degree burn injuries are the most difficult to treat due to permanent damage of the epidermis, dermis, and even the subcutaneous tissue, deep fascia, and muscle. For these injuries, the main goal is to restore the functionality and thickness of the skin, while preventing an immune response and implant rejection. One of the common approaches is grafting. Autologous grafting is advantageous because the tissue is harvested from the patient's body; thus, there is no risk for immune rejection. However, if the injured area is very large, it may be impossible to harvest all of the needed grafts from the patient's body. Consequently, either allografts garnered from either deceased or living human donors or xenografts from different species may be used in the case of unavailable or insufficient allografts. However, because allografts and xenografts usually provide a temporary solution, a second implantation of an autologous graft is necessary to reduce the risk for infection and immune rejection (Janeway et al., 2001). Applying skin grafts over full-thickness wounds usually results in poor skin graft matching, wound contraction, and defected skin tissue remodeling (Sundaramurthi et al., 2014). While some commercially available skin grafts developed for small and chronic wounds have been found to enhance wound healing, they are frail (Dai et al., 2020).

**Figure 7:**
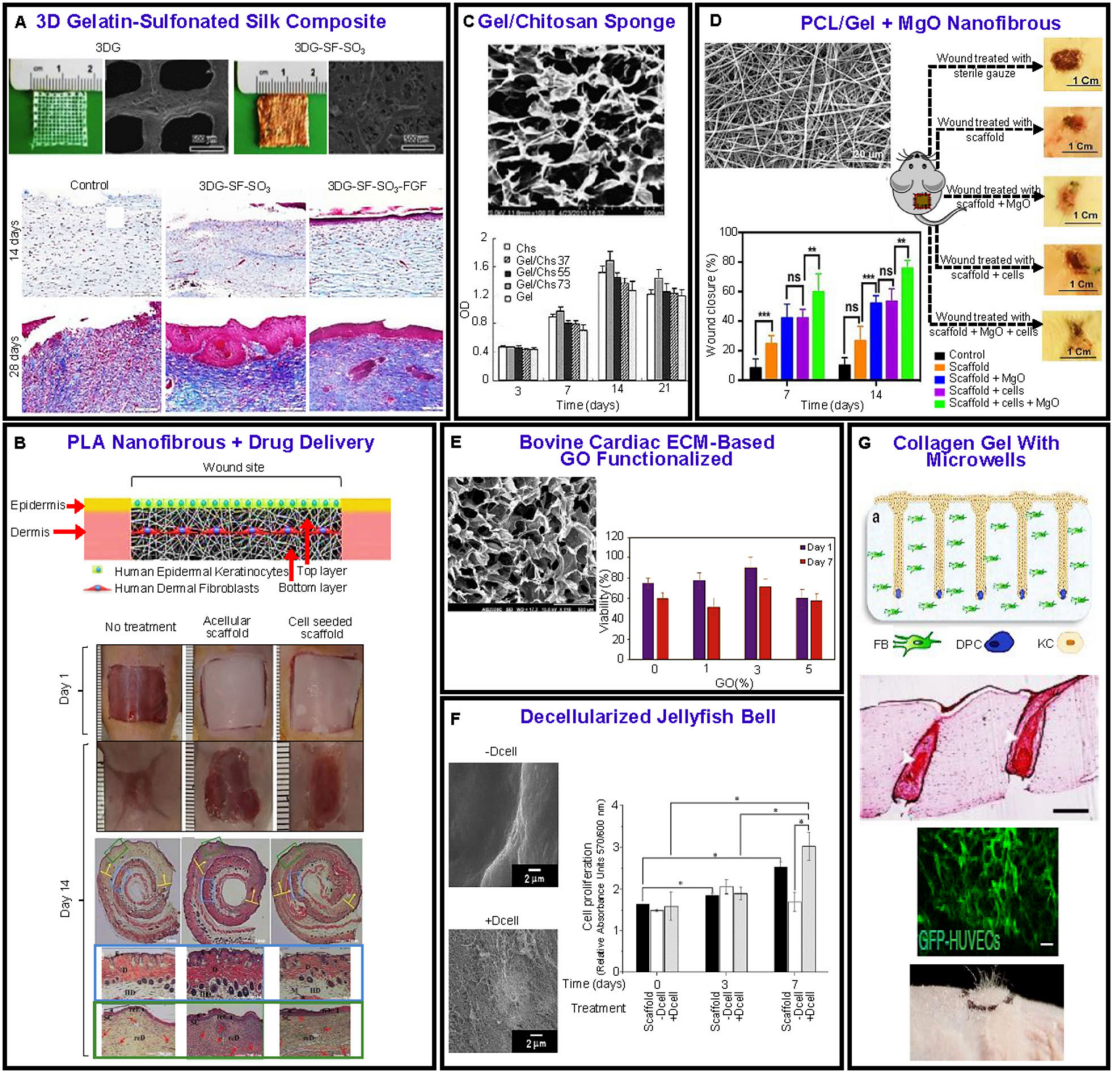
Examples of recent advances in biomaterial scaffolds for skin wound healing. **(A)** Gelatin sulfonated silk scaffolds with FGF-2 growth factor. Masson trichrome staining shows the histology of a repaired wound. The treated samples reveal an increase in collagen content with time relative to the control sample (Xiong et al., 2017). **(B)** Ibuprofen-loaded PLA layered scaffold seeded with fibroblasts and keratinocytes. Wound closure at 1 and 14 days and histological staining of skin samples at 14 days from control wound sites without scaffolds, with acellular scaffold, and cell-seeded scaffold (Mohiti-Asli et al., 2017). **(C)** Gelatin/chitosan freeze-dried scaffold showing enhanced cell viability (Han et al., 2014). **(D)** Hybrid PCL/gel fibrous scaffold loaded with MgO particles showing significantly more wound healing compared to controls (Ababzadeh et al., 2020). **(E)** Porous structure of bovine cardiac ECM-GO functionalized scaffold. Cell viability vs. GO content (Jafarkhani et al., 2020). **(F)** Decellularized (+Dcell) and non-decellularized (–Dcell) jellyfish bell scaffolds. Fibroblast cell proliferation after 0, 3, and 7 days shows a significant difference between +Dcell and –Dcell at 7 days of culture (Fernández-Cervantes et al., 2020). **(G)** Schematic of scaffold and cells seeded, histological staining showing differentiated keratinocyte morphology and graft vascularization, and high follicle density engraftment (Abaci et al., 2018).

Skin scaffolds must supply vital cues for cell viability (i.e., survival, adhesion, and proliferation) and differentiation and support to the growing tissue while loaded with cells, growth factors, and/or medication to prevent inflammation, kill microorganisms, and ultimately reduce wound contraction, skin color mismatch, and other defects in the tissue. Hybrids and composite materials have been used to enhance the scaffold capacity to facilitate the growth of skin tissue. For instance, considering PCL as a synthetic material, hybrid PCL-based fibrous scaffolds electrospun with gelatin using a single solvent have been reported to yield core-shell (coaxial) fibers (Kannaiyan et al., 2019) or uniform fibers (Prado-Prone et al., 2020) and improved cell attachment, proliferation, and matrix formation. Similarly, freeze-dried PCL scaffolds grafted with collagen/chitosan blends by aminolysis (Sadeghi-avalshahr et al., 2020) or blended with collagen/PEG/chitosan (CPCP) (Aghmiuni et al., 2020) demonstrated increased porosity, hydrophilicity, cell infiltration, and antibacterial activity. The CPCP displayed mechanical and chemical properties comparable to those of the decellularized dermal matrix used as control, and when seeded with human adipose-derived stem cells, it resulted in differentiation into both epidermis and dermis keratinocytes, mimicking the human keratinocyte differentiation pattern. Besides seeding scaffolds with stem cells, growth factors have also been directly incorporated into the scaffolds to promote granulation, regeneration of skin-like tissue after implantation in full-thickness defects (Figure 7A), and stimulation of dermal vascularization. The efficacy of the foregoing method has been demonstrated by 3D printed gelatin scaffolds coated with sulfonated silk fibroin and basic fibroblast growth factor 2 (FGF-2) bound by a sulfonic acid (SO_3_) group (Xiong et al., 2017). In another 3D cell printing study of vascularized human skin, the results obtained with a three-layer structure fabricated by consecutive printing hypodermis, vascular channels, dermis, and epidermis using specific materials, cells, and growth factors for each layer (Kim et al., 2019) showed a good prospect for the development of fully functional skin substitutes through the incorporation of skin-related cells.

Skin scaffolds can also be loaded with various medications for local wound release and activation to combat sources of inflammation. PLA electrospun scaffolds loaded with ibuprofen for reducing inflammation and seeded with human epidermal keratinocytes (HEK) and HDF cells in two different layers were found to improve wound contraction and neovascularization (Figure 7B) (Mohiti-Asli et al., 2017). Hybrid materials like PU/PLA scaffolds synthesized by salt leaching and loaded with ciprofloxacin have been proven to inhibit bacterial infections and maintain their physicochemical and mechanical properties for 3–6 months, consistent with skin tissue regeneration requirements (Iga et al., 2020). Scaffolds based on chitosan/gelatin (freeze-dried) loaded with ciprofloxacin hydrochloride have been reported to exhibit decreased inflammatory response and effective cell support and attachment (Figure 7C) (Han et al., 2014). Other antimicrobial agents incorporated in skin scaffolds, such as tetracycline hydrochloride, curcumin, and althea officinalis, have also revealed promising results (Ezhilarasu et al., 2019; Ghaseminezhad et al., 2020).

An effective method to combat inflammation is to add small molecules and/or ligands, such as micro/nanoparticles, self-antigen, and antibodies, which can directly modulate the immune response but not the source of inflammation (Rosique et al., 2015; Gammon and Jewell, 2019; Cleetus et al., 2020). Loading scaffolds with specific types of microparticles and nanoparticles not only can improve the antimicrobial and immune response but may also enhance the scaffold's mechanical performance. To improve scaffold durability and antibacterial activity, skin scaffolds can be loaded with conductive particles or NPs to stimulate cell behavior by applying direct current. However, selecting the size and concentration of NPs is challenging because they may exhibit passivation and cytotoxicity (AshaRani et al., 2009; Jatoi et al., 2019) and the stabilizer may negatively affect the biocompatibility. Importantly, the size of NPs is critical because extremely small particles can penetrate the cell membrane, leading to the formation of vacuoles and, ultimately, premature apoptosis, or aggravate a chronic inflammatory response. Examples of the promising usage of particles in scaffold engineering include fibrous electrospun constructs synthesized from PCL and gelatin blends doped with MgO particles that resulted in 79% wound size reduction when seeded with human endometrial stem cells *in vitro* compared to 11% size reduction accomplished with sterile gauze control (Figure 7D) in addition to improved mechanical properties (Ababzadeh et al., 2020); 3D printed structures of PCL/poly(propylene succinate) copolymer doped with AgNO_3_ demonstrating improved degradation behavior, cell viability, and antimicrobial properties compared to PCL scaffolds (Afghah et al., 2020); electrospun scaffolds of chitosan/gelatin with Fe_3_O_4_ NPs demonstrating enhanced mechanical properties and antibacterial activity (Cai et al., 2016); and scaffolds functionalized with conductive NPs, such as ZnO, Fe_3_O_4_, Au, Ag, and TiO_2_, which enhanced cell proliferation, adhesion, and migration by means of electrical stimulation, or surface roughening by the particles at the scaffold surface (Babitha and Korrapati, 2017; Zulkifli et al., 2017; Kianpour et al., 2020; Radwan-Pragłowska et al., 2020).

Another strategy for skin TE is to augment the cell viability by using decellularized tissue with retained primary ECM constituents. ECM-based materials can be sourced from different organs, such as small bovine intestine (Parmaksiz et al., 2019) and bovine cardiac tissue (Jafarkhani et al., 2020). Functionalized freeze-dried scaffolds consisting of bovine cardiac ECM-based graphene oxide (GO) have demonstrated better mechanical properties and cell viability than decellularized tissue scaffolds (Figure 7E) (Jafarkhani et al., 2020). A marine organism (decellularized jellyfish bell) has also been considered as plausible ECM for engineered skin tissue and found to display intact collagen I that promoted good adhesion and proliferation of HDF cells (Figure 7F), structural stability, good thermal characteristics, and mechanical properties similar to those of collagenous tissues and slightly better than those of human skin (Fernández-Cervantes et al., 2020).

In addition to the materials already mentioned, hydrogel-based skin scaffolds have shown great potential for drug delivery of injectable materials for wound repair because of their intrinsic cellular interaction and biocompatibility (Chaudhari et al., 2016). Several innovations have been reported for injectable hydrogels (Lokhande et al., 2018; Chen et al., 2019; Cheng et al., 2020; Zheng et al., 2020). A notable achievement is an injectable polypeptide-protein hydrogel crosslinked with Ag^+^ demonstrating vascularization, antibacterial activity, wound healing, HF growth, and angiogenesis (Cheng et al., 2020). 3D scaffolds of collagen gel with surface microwells have been developed to control the spatial arrangement of HF (Abaci et al., 2018). Seeding these scaffolds with HDF and human dermal papilla cells (highly specialized mesenchymal cells) and the wells with human dermal keratinocytes led to HF differentiation. In addition, vascularized hair-bearing scaffolds implanted in immunodeficient mice resulted in efficient hair growth. The regeneration of an entire HF from cultured human cells may affect the treatment of different types of alopecia and chronic wounds (Figure 7G) (Abaci et al., 2018). Another approach for generating skin is scaffold-free 3D bioprinting that can deposit layers of different biological materials, called bioinks, including classical biomaterials used to fabricate structural scaffolds (synthetic, natural, or ECM based), different types of cells, growth factors, drugs, DNA, and other bioactive substances. The bioprinting approach has been investigated both *in vivo* and *in vitro* (Pourchet et al., 2017; Kim et al., 2019; Stojic et al., 2019; Jorgensen et al., 2020).

### Brain Scaffolds

Understanding how the human brain works has been the focus of numerous studies. The brain, one of the central nervous system's main parts, is a complex organ that controls vital functions, such as breathing, sleep, sensory processing for vision and hearing, and cognition like reasoning and memory. It is considered to be the crown jewel of the human body and contains more than 100 billion neurons interconnected via branch-like projections of fibrous extensions called axons, which receive and transmit electrochemical signals and contribute in the formation of neuronal-axonal networks. These exquisite networks are complemented by glial cells, recognized as support cells, which are essential for the structural, metabolic, and functional activities of both neurons and axonal tracts. Moreover, the blood brain barrier (BBB), a selective barrier formed by the creation of tight junctions of endothelial cells lining the vasculature in the brain, acts as a physical barrier that regulates the passage of ions, molecules, and cells between the blood and the brain (Abbott et al., 2006). This semi-permeable gate keeps the neural tissue safe and stable by preventing toxins, pathogens, and other harmful substances from entering the brain, while at the same time allowing small gaseous molecules, such as O_2_ and CO_2_, to diffuse freely (Abbott et al., 2010).

When the brain is healthy, it works like a finely calibrated, highly sophisticated machine; however, many serious problems arise when it malfunctions or is injured. During an incidence of traumatic brain injury or a stroke, the sudden energy deficit and hypoxic conditions can cause neurons and glial cells to undergo apoptosis and necrosis, while, concurrently, the BBB triggers the infiltration of immune cells and releases free radicals (Martin et al., 1994). Cell apoptosis and break down of the BBB activate microglia (local inflammatory cells), which express enzymes that reduce the ECM (Graeber and Streit, 2010), leading to the progressive degradation of the mechanical and chemical integrity of the brain tissue, ultimately leading to the formation of a cavity. As a defense mechanism against infarct expansion and further matrix degradation, activated astrocytes undergo astrogliosis, forming a glial scar that acts as a physical and chemical barrier that compartmentalizes the infarct region (Sofroniew and Vinters, 2010). The glial scar isolates the lesion site and prevents the degeneration from proliferating into the healthy tissue. However, the glial scar also inhibits the outgrowth and regeneration of damaged axons, which can cause functional abnormalities or even the death of neurons (Nih et al., 2016).

**Figure 8:**
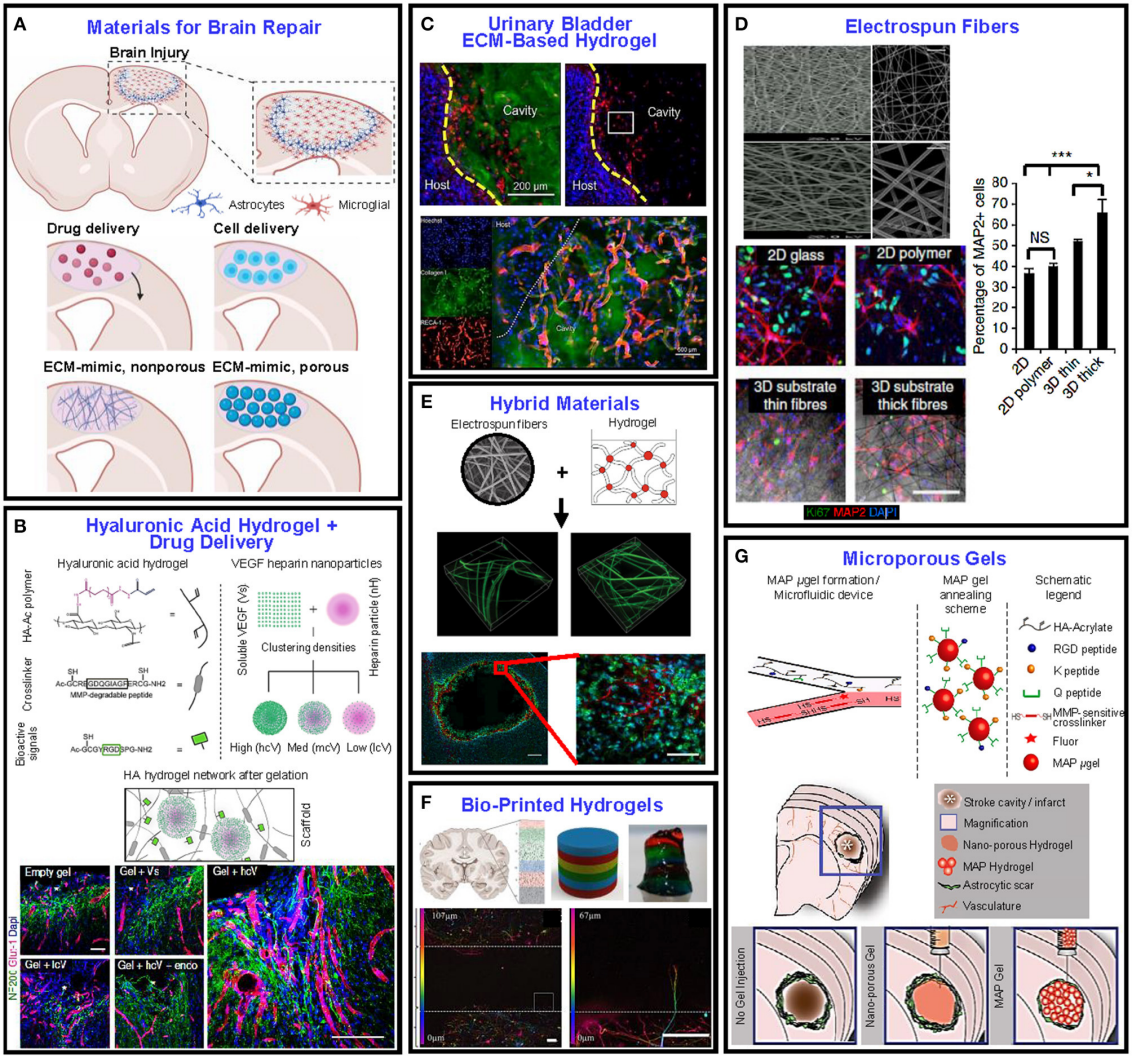
Examples of recent advances in biomaterial scaffolds developed for brain tissue repair after injury. **(A)** Schematic illustration of the pathophysiology after brain injury illuminating the development of astrogliosis and the increase of the microglial density. **(B)** Design of HA with clustered VEGF on heparin NPs. The immunostained images of neurons and vessels show that the gel with high clustered VEGF delivery enhanced angiogenesis and neurogenesis (Nih et al., 2018). **(C)** Urinary bladder-based hydrogel. The immunostained images illustrating ECM hydrogel and cell nucleus show that the hydrogel modulated neuroinflammation and enhanced neurogenesis and angiogenesis (Ghuman et al., 2018). **(D)** Electrospun fibers with different architectures and porosities infiltrated with human-induced neuronal cell populations. Significantly higher numbers of human iN cells expressed microtubule-associated protein 2 in the thick fibrous scaffolds relative to the 2D controls and thin fibrous scaffolds (Carlson et al., 2016). **(E)** Fibers incorporated in hydrogels. When implanted into the striatum, the scaffold displayed higher cellular infiltration and a more loosely defined glial boundary. The images show the tissue-scaffold interface—astrocytes (red), macrophages/microglia (green), and cell nuclei (labeled with DAPI, blue) (Rivet et al., 2015). **(F)** Bio-printed brain-like layer structures (each color represents a different layer). Confocal microscope images of neurons in different layers, colored for the distribution of cells through the *z*-axis, showing an axon penetrating the adjacent layer (Lozano et al., 2015). **(G)** Schematic illustration of the production of hydrogel microparticles in a flow-focusing microfluidic device (Nih et al., 2017). These gels can be injected at the location of interest while preserving their structure.

Most of the efforts focused on brain tissue repair and regeneration have revolved around the delivery of drugs and cells. However, these approaches have not been widely adopted due to daunting challenges. Primary problems related to drug delivery include the constraint of drug diffusion imposed by the BBB and the rapid degradation of the drug once it enters the circulatory system, while challenges associated with cell delivery involve poor survival after delivery and reduced integration into the host tissue. Biomaterial scaffolds play a central role in contemporary strategies for developing viable solutions that can mitigate some of the above limiting issues and, consecutively, facilitate brain tissue regeneration, reconstruction, and reconnectivity of neuronal-glial networks following degeneration due to brain injury or disease (Zhong and Bellamkonda, 2008; Khaing and Schmidt, 2012; Khaing et al., 2014) (Figure 8A). The fragile nature of the brain tissue, which is soft (its elastic modulus ranges from 1 to 14 kPa) and anisotropic (its ECM consists of collagen type IV, HA, fibronectin, laminin, and proteoglycans), in conjunction with its confined space are specific design criteria for biomaterials intended for brain tissue repair (Tuladhar et al., 2018). The ultimate goal of such biomaterial scaffolds is to facilitate functional recovery of the brain tissue by promoting regeneration, elicit plasticity, and drive neurogenesis. Scaffolds for brain tissue repair have been used to perform localized drug and cell therapies to the injury site, provide mechanical support to the surrounding tissue, and guide the growth of neural axonal networks.

Hydrogels have attracted considerable interest as scaffolds for brain repair because they can form ECM-mimetic architectures and may serve as a platform for minimally invasive drug and cell delivery directly at the injury site (Lee and Mooney, 2001). When designing injectable hydrogels for the post-injury environment of the brain, the physical properties that must be taken into account are the porosity, chemical composition, and mechanical characteristics. Because the brain tissue is more sensitive to stress effects than other tissues (Liao et al., 2008; Pettikiriarachchi et al., 2010), the stiffness of biomaterials implanted in the brain must be similar to that of the host tissue, because stiffer materials usually lead to gliosis and inflammation, whereas softer materials result in implant instabilities and failure. Hydrogel scaffolds can provide structural support to the surrounding tissue, can be loaded with drugs and growth factors, and can also serve as cell transplantation vehicles to deliver neural progenitor cells (NPCs), hence providing an important mechanism for rebuilding a functional neuronal network after brain injury (Orive et al., 2009; Erning and Segura, 2020). Natural hydrogels, such as HA (Tian et al., 2005; Wang et al., 2011; Mittapalli et al., 2013), collagen (Guan et al., 2013), chitosan (Yang Z. et al., 2010), agarose (Jain et al., 2011), and methylcellulose (Cooke et al., 2011; Austin et al., 2012), have been used in brain injuries and their capability to reduce inflammation, guide axonal growth, and promote angiogenesis has been assessed (Kornev et al., 2018).

Localized drug delivery by injectable hydrogels outperforms traditional drug delivery approaches because hydrogel biomaterials provide a delivery platform that demonstrates superior spatiotemporal control (Zhu et al., 2015). The encapsulation of drugs and growth factors into particulates incorporated in the bulk of implantable hydrogels reduces inflammation, improves neurogenesis, and results in minimal tissue damage. For instance, hyaluronan-methylcellulose encumbered with PLGA NPs loaded with drug and/or growth factors are classified as composites with highly tunable and sustainable delivery profiles. The injection of this system above a stroke-induced lesion of a mice caused the local and sustained delivery of brain-derived neurotrophic factor to increase neuroplasticity and decrease the lesion cavity and neuron loss (Obermeyer et al., 2019). Moreover, hydrogel biomaterials used in brain injuries can be loaded with anti-inflammatory drugs to control inflammatory reaction. For example, biodegradable gelatin microspheres loaded with anti-inflammatory drug were used to protect the encapsulated drug from inflammation-derived degradation after administering it to the injury site (Jin et al., 2014). The direct transport of angiogenic agents, such as vascular endothelial growth factor (VEGF), can cause further brain damage by augmenting BBB breakdown and inducing immature and disorganized vessel formation (Aday et al., 2017). To overcome this barrier, hydrogel biomaterials have been used to guide the delivery of VEGF, overcoming difficulties with repetitive local injections and the short half-life of VEGF. An HA-based hydrogel crosslinked *in situ* with MMP degradable peptide and decorated with RGD adhesive motifs co-injected with VEGF clustered on heparin NPs into an injury cavity suppressed inflammation and enhanced angiogenesis, neurogenesis, and axonal growth (Figure 8B) (Nih et al., 2018).

Hydrogel scaffolds can also direct the movement and differentiation of neural stem and progenitor cells into specific cell types, thus contributing to the recovery process. Because cell survival during transplantation is critical, optimizing the hydrogel properties (e.g., stiffness, gelation time, porosity, etc.) is very important (Adil et al., 2017). In a study that used an *in situ* formed HA/peptide hydrogel to transplant NPCs derived from stem cells into the infarct cavity of a stroked mice, the key factors that regulated cell survival during transplantation were found to be the injection speed and the needle gauge during the injection and gelation process (Lam et al., 2014). It was also observed that cells encapsulated in the hydrogel demonstrated enhanced NPC differentiation to a neuronal phenotype (neuroblasts) compared to cells transplanted without using the hydrogel as a vehicle. Additionally, hydrogels exhibiting mechanical properties close to those of native tissue, which were decorated with natural ECM molecules and loaded with tissue-relevant factors and topography cues, were found to direct the differentiation of the transplanted cells (Khaing et al., 2014). Another strategy for brain repair is to prepare scaffolds from decellularized tissue. ECM hydrogels have been used in endogenous brain repair after injury (Ghuman et al., 2016; Wu et al., 2017). ECM-based hydrogels can be sourced from different organs, such as the urinary bladder matrix (Freytes et al., 2008), peripheral nervous system (Prest et al., 2018), and brain (Crapo et al., 2012). For example, a porcine-derived urinary bladder ECM matrix was used to form injectable ECM hydrogels for delivery in an ischemic stroke mouse model (Figure 8C) and the results showed that low concentration and optimal biodegradation rate of the hydrogel improved immunomodulation, angiogenesis, and neurogenesis (Ghuman et al., 2018).

Hydrogels are often mentioned in TE approaches for brain repair due to their biocompatibility and inherent bioactive properties. However, synthetic polymeric scaffolds have also been utilized because their bulk properties and degradation rates can be more easily controlled (Kweon et al., 2003; Chew et al., 2007; Mahumane et al., 2018). Most of the studies considered synthetic polymeric scaffolds as potential carriers of drugs, cells, and growth factors to the injury site. The architecture, fiber alignment, porosity, and topography are critical when designing synthetic polymeric scaffolds for brain repair. The composition of a polymer scaffold governs the early interfacial phenomena that control cell attachment, whereas the polymer architecture (fibrous or porous) directs the long-term cellular behavior. For example, studies with electrospun tyrosine-derived polycarbonate substrates with thin and thick fibers and different porosities (Figure 8D) demonstrated that larger voids in the substrates with thick fibers enhanced the volumetric permeability for cellular infiltration, promoted stem cell neuronal reprogramming and neural network establishment, and supported neuronal engraftment in the brain; however, the substrates with thin fibers did not show the same results (Carlson et al., 2016). Although the nature of the brain tissue promulgated the development of injectable hydrogels for implementation at the site of injury, implantable polymeric scaffolds have also been used mostly together with hydrogels to form hybrid materials that can synergistically provide the physical and chemical cues for instigating cell migration into the hybrid matrix (Bosworth et al., 2013; Rivet et al., 2015). *In vitro* studies with the foregoing hybrid materials (Figure 8E) have provided insight into fundamental problems regarding the use of biomaterials for brain tissue repair (Hopkins et al., 2015). In this context, recent advancements in 3D printing and the concomitant use of bio-inks and cells can be instrumental to the development of brain-like constructs consisting of discrete cell layers that can be used *in vitro* to further explore cellular behavior and elucidate critical biomaterial issues pertaining to brain tissue injury and repair (Figure 8F) (Hopkins et al., 2015; Koffler et al., 2019).

Biomaterial scaffolds for brain tissue repair have been used to improve the delivery of drugs, growth factors, and cell transplantation. Recently, biomaterials that can promote tissue repair and regeneration without the need to transport cells or other therapeutics have provided a powerful approach to brain tissue scaffolding. Injectable porous hydrogels, referred to as hydrogel microparticles (HMPs), have arisen as promising biomaterials for brain repair and biomedical engineering (Figure 8G) (Griffin et al., 2015; Nih et al., 2017; Daly et al., 2019). These porous hydrogels exhibit shear-thinning behavior, are inherently modular, and because of the interstitial space between the packed HMPs they possess significant porosity that is important for tissue repair and reduced inflammation not only in the brain but also in other organs (Madden et al., 2010; Tokatlian et al., 2014). In a specific example, an HA microparticle hydrogel injected in a stroke cavity reduced astrogliosis and modulated neuroinflammation, illuminating the critical role of biomaterial design in tissue repair (Christman, 2019; Sideris et al., 2019).

## Outlook and Future Perspectives

**Figure 9:**
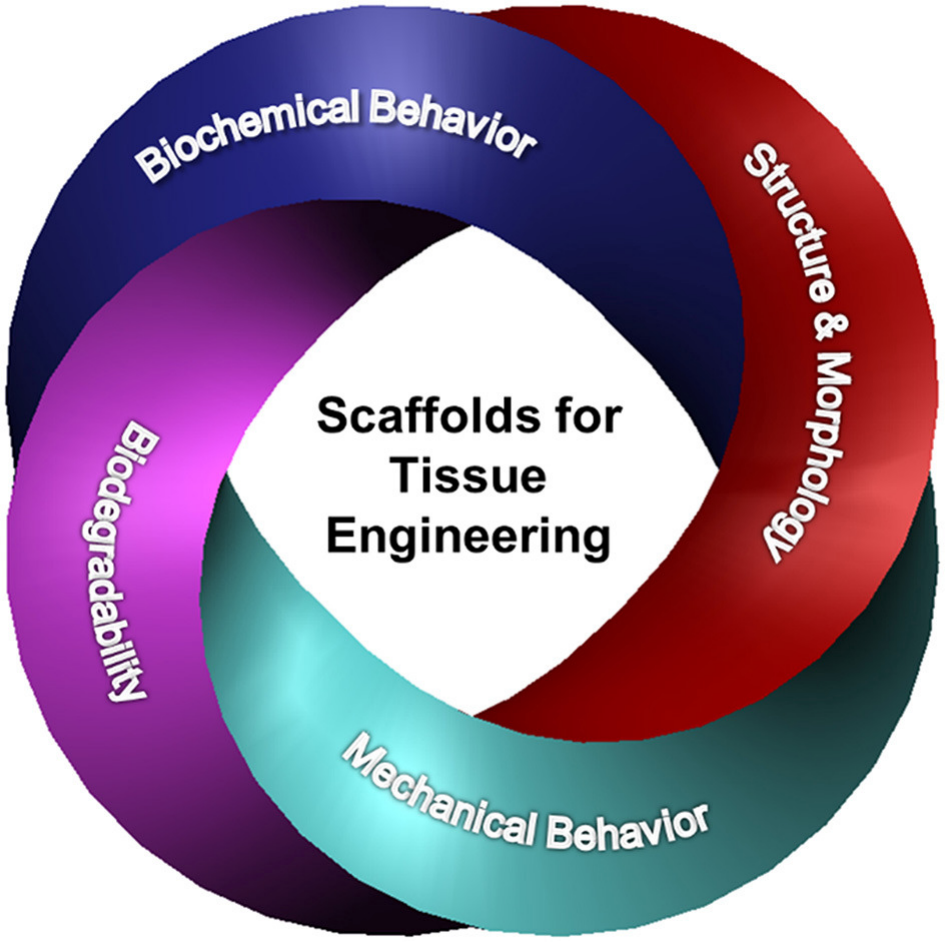
Synergy between biochemical behavior, structure and morphology, biodegradability, and mechanical behavior of scaffolds activates cellular behavior, controls cell fate, and drives TE advancement.

The foregoing synopsis elucidates some of the most important accomplishments, the current demands, and the existing challenges regarding biomaterial scaffolds used in various TE applications. The development of such functional constructs for augmenting or replacing native tissues lies at the crossover of biology, medicine, materials science, and engineering. Numerous new strategies have been advanced to fabricate highly tunable and functional scaffolds. However, despite the outstanding progress achieved to date, there are still numerous challenges that must be overcome before scaffold-based TE technologies can attain broad clinical usage and commercialization. Several design considerations that are undistinguishably linked to the formation, function, and implant location of native tissue must be addressed to further advance scaffold engineering. The chemical, morphological, and mechanical properties of scaffolds must be tuned to optimize interactions with the cells and the surrounding tissue, whereas the biodegradation rate must be controlled to preserve the scaffold's integrity until the maturity of the growing tissue. Scaffolds should be designed with an appreciation of the bio-chemo-mechanical properties of the native tissue and the complex mechanisms that control cell interactions. Furthermore, the composition and micro/nanostructure of the ECM of different tissues vary significantly. Therefore, recapitulating the biological roles of the native tissue through a synthetic construct should recognize the structure architecture of the tissue of interest and its biological role. Clearly, there are no universal design criteria that fit all of the tissue characteristics. Because native tissues are characterized by a wide range of functions and compositions, the same scaffold materials cannot be used for all tissues. When selecting and developing new materials for TE, it is imperative to simultaneously consider the complex biochemistry, morphology, mechanical behavior, and biodegradation characteristics of the scaffold (Figure 9).

The interactions between the native cells and the scaffold promote distinct cell phenotype changes and affect the regeneration of the tissue. While these effects were initially attributed to interactions at the cell-scaffold interface, cumulative evidence suggests that the 3D structure architecture of the scaffold plays a crucial role. The biochemical behavior of the scaffold dictates whether the cells will proliferate, change the types of ligands and receptors present at their surfaces, or change function. If the scaffold does not possess the right cues, the cells may detach from the matrix and undergo apoptosis. In view of the scaffold-dependent cell behavior, it is imperative to characterize scaffold materials for both short- and long-term cell phenotype effects. Strategies to control and recap cell-material interactions should account for the effect of scaffold structure across multiple length scales.

The necessity for scaffolds that can deliver oxygen and nutrients while removing cell products and waste has been a central theme in multiple TE efforts. Increasing the cellular density in a scaffold to mimic the density of the native tissue may produce a hypoxic core that usually leads to cell dysfunction and death. It is vitally important that a blood supply is within the vicinity of the cells so that they can sense the physiological cues and access the oxygen and nutrients that are necessary for their long-term viability (Lokmic and Mitchell, 2008). In native tissues, the cells are in proximity of a few hundred of micrometers from a flowing blood supply, because mass transport is insufficient for a larger distance (Folkman and Hochberg, 1973). Therefore, the fabrication of viable tissue-engineered constructs of a size larger than the latter distance range appears to be inconceivable in the absence of a natural vascular network, including vasculogenesis and angiogenesis. Vasculogenesis generates new vascularization in the absence of preexisting blood vessels, whereas angiogenesis is defined as the formation of new vessels from an existing vascular network (Auger et al., 2013). The vascular networks play a pivotal role in the survival of cells; however, their quality is more critical than their quantity. When aiming for vascular networks in engineered tissues, the blood perfusion and distribution over a tissue volume is more important than the number of vascular structures. An optimal vascular network must be highly organized and include capillaries, venules, and arterioles to sufficiently supply the cells with oxygen and nutrients. Besides vascular organization, other key parameters, such as perfusability and barrier function, must also be taken into account. In addition, there is a need for an optimum balance between vascular organization in engineered tissues before implantation and vascular remodeling after implantation. An initial degree of vascular organization is necessary for supplying the cells with nutrients, whereas vascular remodeling after implantation is critical to adapting to the post-implantation environment. Likewise, while it is clear that endothelial cells are needed to form the lining of the structures, it is essential to investigate which combination of cell types (i.e., macro/microvascular and progenitor based) can lead to the formation of an optimal vascular network for a specific tissue (Rouwkema and Khademhosseini, 2016). Therefore, a scaffold intended for completely tissue-engineered organs requires spatial control over different tissue layers and types and the incorporation of perfused vascularization to promote tissue health and recapitulate tissue function.

Despite several decades of intense research, the widespread use of TE remains encumbered not only due to the engineering design constraints highlighted in this article but also because of several regulatory hurdles. Concerns regarding cell seeding, poorly defined degradation products, and the need for extensive pre-clinical data increase both the time and the cost to achieve regulatory approval for clinical translation of scaffold-based TE technologies. Furthermore, most of the conducted studies have relied on scaffold implantation in animal models like rodents and rabbits. This raises many concerns about the performance of engineered scaffolds in the human body due to interspecies variability between animal models and human immunology (Mestas and Hughes, 2004; Seok et al., 2013). Therefore, more efforts must be devoted to the implementation of animal models that closely resemble human physiology, and pre-clinical data of the scaffold efficacy should be acquired from multiple animal models across various species. Finally, it is critical to understand that the majority of TE approaches combine cells seeded into scaffolds with signaling cues and chemical functionalities or drugs, thereby rendering the regulatory pathways more complex and necessitating the use of costly methods to demonstrate implant safety.

As the medical challenges become more abundant, the need to improve the quality of life for patients worldwide through TE innovation is becoming more evident. The limited clinical success of TE thus far signifies the enormous scientific, medical, and financial challenges faced in this field and underlines the importance of collaboration among scientists, engineers, and physicians more than ever. It is critically important that the requirements of medical groups are aligned with the responses of biomaterial groups and that a strong commitment exists among financial partners, government agencies, and corporations to enable innovative advances in scaffold engineering for a wide range of TE applications.

## Author Contributions

MEM and KM collected most of the pertinent literature, created the figures, and wrote the first draft of the paper. KK defined the scope of the entire work, supervised the work of MEM and KM, examined the accumulated results, and edited the final manuscript. All authors contributed to the article and approved the submitted version.

## Conflict of Interest

The authors declare that the research was conducted in the absence of any commercial or financial relationships that could be construed as a potential conflict of interest.
